# Fringe-positive Golgi outposts unite temporal Furin 2 convertase activity and spatial Delta signal to promote dendritic branch retraction

**DOI:** 10.1016/j.celrep.2022.111372

**Published:** 2022-09-20

**Authors:** Hsun Li, Hsin-Ho Sung, Yi-Chun Huang, Ying-Ju Cheng, Hsiao-Fong Yeh, Haiwei Pi, Edward Giniger, Cheng-Ting Chien

**Affiliations:** 1 Institute of Molecular Biology, Academia Sinica, Taipei 11529, Taiwan; 2 Taiwan International Graduate Program in Interdisciplinary Neuroscience, National Yang Ming Chiao Tung University and Academia Sinica, Taipei 11529, Taiwan; 3 Neuroscience Program of Academia Sinica, Academia Sinica, Taipei 11529, Taiwan; 4 Department of Biomedical Sciences, College of Medicine, Chang Gung University, Tao-Yuan 33302, Taiwan; 5 National Institute of Neurological Disorders and Stroke, National Institutes of Health, Bethesda, MD 20892, USA; 6 Lead contact

## Abstract

Golgi outposts (GOPs) in dendrites are known for their role in promoting branch extension, but whether GOPs have other functions is unclear. We found that terminal branches of *Drosophila* class IV dendritic arborization (C4da) neurons actively grow during the early third-instar (E3) larval stage but retract in the late third (L3) stage. Interestingly, the Fringe (Fng) glycosyltransferase localizes increasingly at GOPs in distal dendritic regions through the E3 to the L3 stage. Expression of the endopeptidase Furin 2 (Fur2), which proteolyzes and inactivates Fng, decreases from E3 to L3 in C4da neurons, thereby increasing Fng-positive GOPs in dendrites. The epidermal Delta ligand and neuronal Notch receptor, the substrate for Fng-mediated O-glycosylation, also negatively regulate dendrite growth. Fng inhibits actin dynamics in dendrites, linking dendritic branch retraction to suppression of the C4da-mediated thermal nociception response in late larval stages. Thus, Fng-positive GOPs function in dendrite retraction, which would add another function to the repertoire of GOPs in dendrite arborization.

## In brief

Li et al. report a stage-specific dendritic branch retraction in *Drosophila* nociceptive neurons. A subset of Golgi outposts in dendrites carrying the O-glycosylation enzyme Fringe localizes at distal branches to promote branch retraction through non-canonical Notch signaling. The proprotein convertase Furin 2 proteolyzes Fringe to inhibit its localization at Golgi outposts.

## Graphical abstract

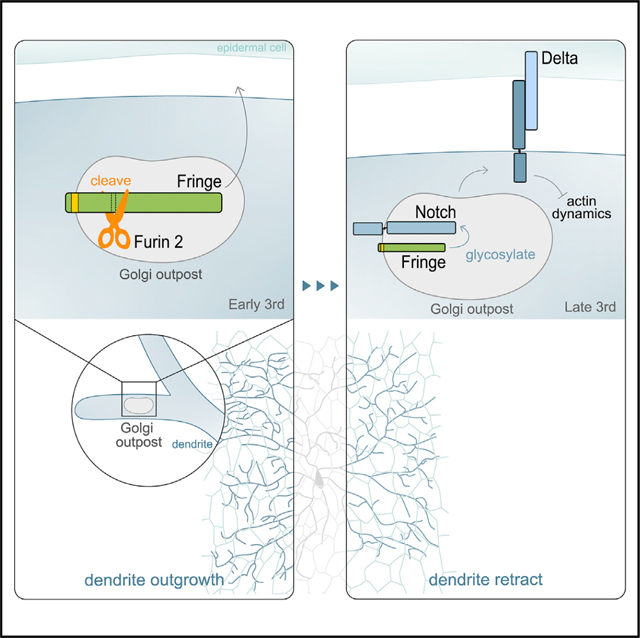

## INTRODUCTION

Dendrites act as the signal-receiving end of neurons and possess a stereotypical and complex morphology. Stage-specific dendritic arborization is crucial for circuit formation and brain function, requiring a balance between growth and pruning. Dendritic growth is dynamically contributed by branch formation and elongation. During development, dendrites of many types of neurons are pruned (Furusawa and Emoto, 2020). Terminal branches undergo retraction and elimination, whereby unstable or unnecessary ones are removed. Dendrites also undergo systematic reduction during specific developmental stages, such as postnatal stages, before the dendrite attains maturity (Conel, 1939; Huttenlocher, 1979).

Large-scale pruning of dendrites occurs in the *Drosophila* class IV dendritic arborization (C4da) neurons during metamorphosis (Kuo et al., 2005; Williams and Truman, 2005). Trimming of terminal branches associated with retarded actin dynamics in C4da neurons has also been shown at the late third-instar larval stage (Nithianandam and Chien, 2018). Although the mechanism underlying large-scale removal of an entire dendritic tree has been studied extensively for the pupal stage (Yu and Schuldiner, 2014), how trimming of terminal branches is regulated and executed remains unclear.

Golgi outposts (GOPs) are discrete Golgi-like organelles distributed throughout the dendritic arbor and play a pivotal role in dendritic arborization (Ehlers, 2013; Jan and Jan, 2010). Within dendritic arbors, the GOPs act in modifying synthesized proteins and lipids from the endoplasmic reticulum (ER) (Hanus and Ehlers, 2008). In cultured mammalian hippocampal CA1 neurons, disruption of ER-to-Golgi or post-Golgi trafficking causes aberrant GOP distributions and reduces apical dendrite complexity (Horton and Ehlers, 2003; Horton et al., 2005). Moreover, mutations of genes functioning in the Golgi-dependent secretory pathway, and polyglutamine toxicity- or dipeptide repeat protein toxicity-induced GOP loss, cause dendritic growth deficiency in C4da neurons (Chung et al., 2017; Park et al., 2020; Ye et al., 2007). Also, the subset of stationary GOPs colocalizes with the Leucine-rich-repeat kinase protein, restricting dendrite growth (Lin et al., 2015). The subset of GOPs that associate with the γ-tubulin-containing microtubule organization center induces local microtubule polymerization (Ori-McKenney et al., 2012). Thus, dendritic GOPs are likely heterogeneous in content and multifunctional.

Golgi complexes within single cells could be heterogeneous, as reflected by diversified localizations and the various functions of Golgi proteins (Wei and Seemann, 2010). The *Drosophila* imaginal disc cells contain many small and dispersed Golgi fragments (Kondylis et al., 2001). Interestingly, these Golgi fragments are dedicated to distinct signaling activities. For example, Fringe (Fng), a glycosyltransferase involved in building the O-glycan chains attached to the extracellular domain of the Notch (N) receptor, mainly colocalizes with the nucleotide-sugar transporter Frc (Yano et al., 2005). However, Fng rarely colocalizes with Rhomboid (Rho) and Sulfateless (Sfl), which are also Golgi-localized and related to other signaling pathways (Sturtevant et al., 1993; Toyoda et al., 2000). Therefore, we investigated whether GOPs are also heterogeneous in dendrites. In C4da neurons, we observed that Fng localized to dendritic GOPs with a spatiotemporal pattern. Interestingly, the Fng distribution in distal dendritic regions was correlated with terminal branch retraction at the late larval stage. We examined how Fng-localized GOPs are regulated spatiotemporally and how Fng modulates non-canonical N signaling in dendritic branch retraction.

## RESULTS

### A transition in dendritic branch dynamics of C4da neurons during larval stages

**Figure 1.**
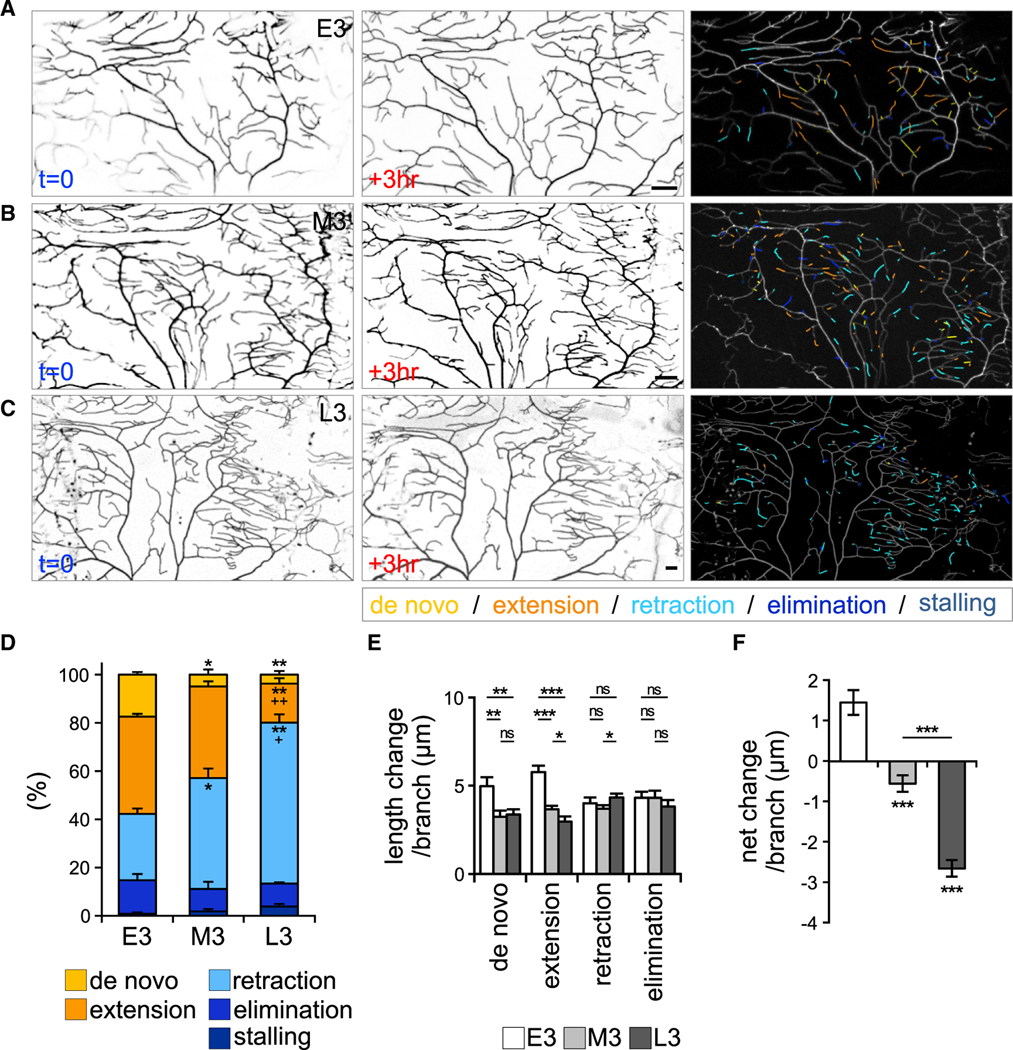
Dynamics of terminal branches of C4da dendrites in E3, M3, and L3 stages (A–C) Three-hour-span images of *ppk-CD4-tdTom*-labeled C4da neurons in (A) E3, (B) M3, and (C) L3 stages. Changes to the branches within the 3 h are color-coded (right). (D) Percentages of branches in different dynamic categories averaged from three neurons in each stage. (E) Length changes per branch in different dynamic categories in E3 (n = 422), M3 (n = 499), and L3 (n = 509) stages. (F) Net change per branch in the three stages. Bar graphs represent the mean ± SEM. Statistical significance was determined by Student’s t test, comparing with E3 or comparing groups indicated by lines, and shown as *p < 0.05, **p < 0.01, ***p < 0.001, or ns for not significant, and in (D), ^+^p < 0.05 and ^++^p < 0.01 comparing L3 to M3. Scale bars, 20 μm.

Dendritic terminal branches of C4da neurons are reduced in the late third-instar larval stage, prior to the large-scale pruning in the pupal stage (Parrish et al., 2009; Williams and Truman, 2005). Therefore, we examined the terminal branch dynamics profile for three third-instar larval stages (Figures 1A–1C) to understand how it contributes to overall reduced arborization. In the early third-instar (E3) stage, more dendritic branches underwent *de novo* formation and extension than retraction and elimination, leading to increased overall branch length (Figures 1A and 1D–1F). Subsequently, in the mid-third-instar (M3) stage, reduced *de novo* and extending branches and increased branch retraction were seen, bringing a slight reduction in overall length (Figures 1B and 1D–1F). After the transition to the late third-instar (L3) stage, we observed a higher increase in branch retraction and reductions in *de novo* and extending branches, causing a striking reduction in overall branch length (Figures 1C and 1D–1F). Our analysis of branch dynamics in three consecutive stages suggests developmental transitions in C4da dendrites, with a propensity for outgrowth in E3, through a relatively balanced state in M3, to a withdrawal state in L3.

### Constant GOP distribution in C4da dendrites during three larval stages

Dendritic GOPs are thought to be involved in dendritic dynamics and arborization (Lin et al., 2015; Ori-McKenney et al., 2012; Ye et al., 2007). We wondered if the GOP distribution patterns differ in the E3, M3, and L3 stages, contributing to different branch dynamics. We analyzed the overall distribution of GOPs labeled with GFP-fused α-mannosidase II (ManII-GFP) in CD4-tdTom-labeled C4da dendrites (Figures S1A and S1B). First, we found that the GOP numbers per neuron increased gradually from E3 to L3 (Figure S1C), while the dendritic arbor size expanded dramatically (Figures S1D and S1E). When we compared the GOP radial distributions, only the difference between E3 and L3 was significant. The averaged median radii were 70 (E3), 102 (M3), and 125 μm (L3), highlighting the widening GOP distributions at later larval stages (Figure S1F). Considering that dendritic arbor growth would contribute to the widening GOP distributions, we conducted normalization for the GOP distributions (see STAR Methods). Interestingly, neither the normalized GOP distributions nor the averaged median radii were different between any two stages of E3, M3, and L3 (Figure S1G). Thus, the almost equivalent GOP distributions are unlikely to account for the distinct branch dynamics in these three stages (Figure 1).

### Restricted distribution of Fng-positive GOPs in dendrites at early larval stages

We hypothesized that GOPs could be heterogeneous and that subsets of GOPs could function specifically in branch retraction. The Golgi-resident proteins Fng, Rho, and Sfl localize at distinct Golgi units in wing-disc cells (Yano et al., 2005). Therefore, we tested whether they also exhibit distinct localizations in C4da dendrites by generating transgenes of Fng-GFP, GFP-Rho, and Sfl-GFP with GFP exposed in the Golgi lumen. We first confirmed that overexpressed Fng-GFP, GFP-Rho, and Sfl-GFP localized at subsets of Golgi fragments in wing-disc cells (Figures S2A–S2C). We then examined their localization in C4da neurons and found that Fng-GFP signals localized at 69% ± 1.4% of somatic Golgi fragments marked by CFP-Golgi, and GFP-Rho at 43% ± 0.5%, whereas Sfl-GFP signals were diffuse (Figures S2D–S2F). Fng-GFP and GFP-Rho also formed discrete puncta in dendrites (Figures S2G and S2H). However, Sfl-GFP was almost undetectable in dendrites (Figure S2I).

**Figure 2.**
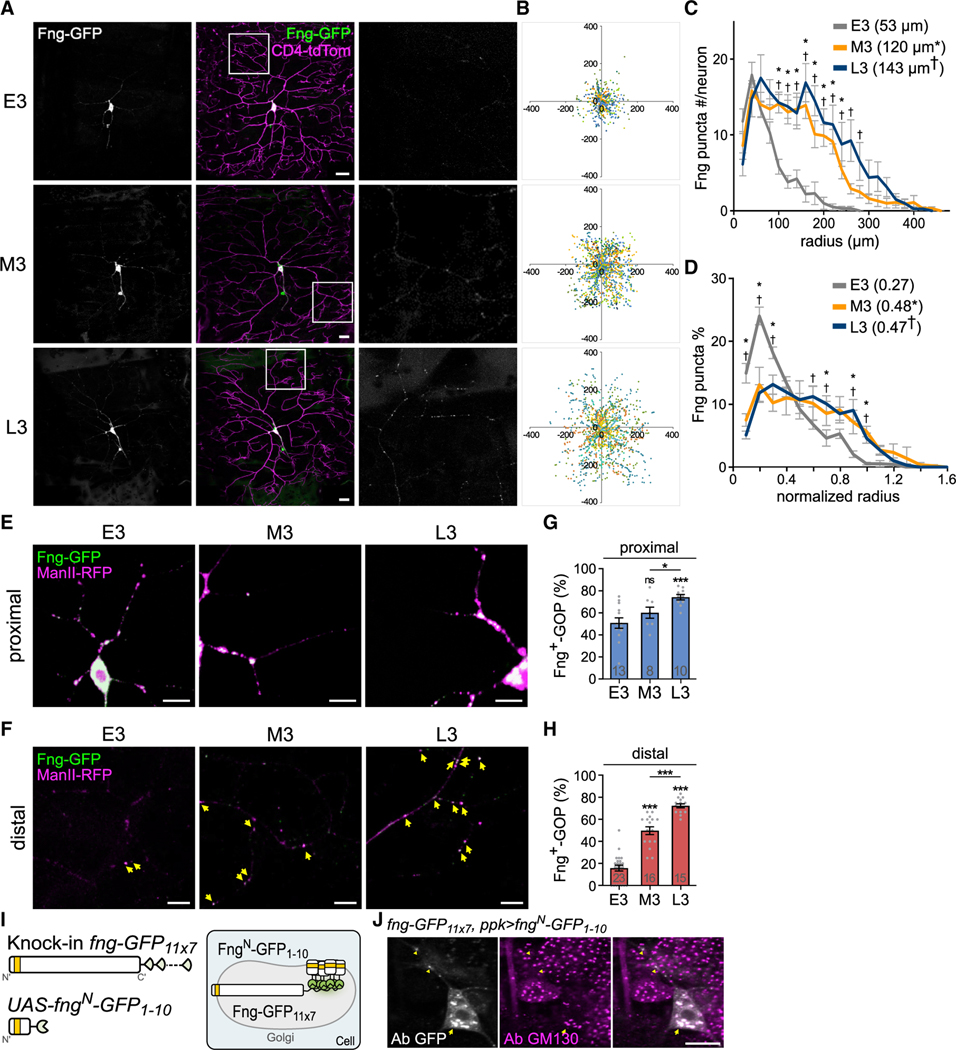
Enhanced Fng distribution in distal dendritic regions of C4da neurons at the M3 and L3 stages (A) Distribution of *ppk-GAL4*-driven Fng-GFP in CD4-tdTom-labeled C4da dendrites in the E3, M3, and L3 stages. Boxed areas are magnified in the right column to show Fng-GFP puncta. (B) Scatterplots of the Fng-GFP puncta distributions from C4da neurons (each coded with a different color); the x and y axes (μm) represent anteroposterior and dorsoventral directions. (C and D) Concentric distribution curves for Fng-GFP puncta in E3 (n = 10), M3 (n = 10), and L3 (n = 8) stages show (C) numbers of puncta in radial intervals or (D) percentages of puncta in the normalized radial intervals. Statistical significance was determined by two-way ANOVA with Bonferroni *post hoc* test. Averaged median radii are shown in parentheses, and statistical significance was determined by Student’s t test. *p < 0.05 comparing E3 and M3 and †p < 0.05 comparing E3 and L3. (E and F) Co-overexpression of Fng-GFP and ManII-RFP by *ppk-GAL4*, revealed in (E) proximal and (F) distal dendritic regions. Fng^+^-GOPs are indicated by yellow arrows in distal regions. (G and H) Fng^+^-GOP percentages in (G) proximal and (H) distal dendritic regions in each stage. Data and bar graphs represent the mean ± SEM. Statistical significance was determined by Student’s t test, and shown as *p < 0.05, ***p < 0.001, and ns for not significant. (I) Schematics show detection of endogenous Fng with knockin *fng-GFP*_*11×7*_ and GAL4-driven *UAS-fng*^*N*^*-GFP*_*1–10*_, including the transmembrane domain (yellow), and the reconstituted Fng-GFP_11×7/1–10_ (green) locates in the Golgi lumen. (J) With *ppk-GAL4*-driving *UAS-fng*^*N*^*-GFP*_*1–10*_ expression in the *fng-GFP*_*11×7*_ knockin larvae, reconstituted Fng-GFP_11×7/1–10_ was detected by GFP antibody immunostaining (Ab GFP, white) and localized at Golgi structures as revealed by GM130 immunostaining (magenta) in C4da dendrites (arrowheads) and soma (arrows). Scale bars, 20 μm in (A) and 10 μm in (E, F, and J).

We focused our study on Fng, since we found differential distributions of Fng-GFP puncta in C4da neurons at different larval stages. To characterize it, we examined the Fng-GFP punctum distribution in C4da dendrites in the E3, M3, and L3 stages (Figures 2A and 2B). Strikingly, the Fng-GFP punctum numbers significantly increased from E3 to M3 and from M3 to L3 (Figure S3A). Furthermore, the Fng-GFP punctum distribution curve in E3 declined sharply upon reaching a peak adjacent to the soma, but the M3 and L3 curves declined gradually. The median radii also increased significantly from early to late stages (Figure 2C). We further analyzed the normalized distributions in these three stages and found that the E3 curve was distinctive, with a smaller median radius (Figure 2D). These analyses indicate a propensity for expanded distributions of Fng-GFP puncta in the medial and distal dendritic regions in the M3 and L3 stages, markedly different from the constant GOP distribution at all three stages (Figure S1).

To examine whether the Fng-GFP puncta localize at GOPs, we co-overexpressed Fng-GFP with Golgi-localized ManII-RFP in C4da neurons and quantified the Fng-GFP-positive GOPs (Fng^+^-GOPs) in dendrites (Figures 2E and 2F). We found that Fng^+^-GOP percentages in proximal dendritic regions increased slightly from E3 to M3 and from M3 to L3 (Figure 2G). Instead, the increases in Fng^+^-GOP percentages from E3 to M3 and from M3 to L3 in distal dendritic regions were more significant (Figure 2H), indicating a preferential distal localization for increased Fng^+^-GOP at later larval stages.

To further confirm that the endogenous Fng protein localizes in dendrites, we utilized the system developed for detecting low-level proteins in specific cell types by reconstituting and amplifying GFP signals (Kamiyama et al., 2021). In brief (see STAR Methods for details), the knockin *fng-GFP*_*11×7*_ allele, carrying seven repeats of split GFP_11_ at the C terminus of *fng*, was coupled with the GAL4-driven *UAS-fng*^*N*^*-GFP*_*1–10*_. The N terminus of Fng (Fng^N^) includes the putative Golgi localization signal and the transmembrane domain (Figure 2I). With *ms1096-GAL4-*driving Fng^N^-GFP_1–10_ expression in the wing discs, the reconstituted Fng-GFP_11×7/1–10_ signals were detected in the dorsal pouch (Figure S3B, top), the documented Fng expression domain (Irvine and Wieschaus, 1994). The reconstituted GFP signals also localized at the Golgi fragments (Figure S3B, bottom). As a negative control, we failed to reconstitute GFP signals from coupling Fng-GFP_11×7_ with the secreted protein BiP^SP^-GFP_1–10_ (Figures S3C and S3D).

We then tested the reconstitution of Fng-GFP_11×7/1–10_ in C4da neurons with the *ppk-GAL4* driver. We found that Fng-GFP_11×7/1–10_ signals localized at Golgi fragments in cell bodies and GOPs in dendrites of C4da neurons (Figure 2J). In the control, no apparent GFP signals could be detected in C4da neurons expressing BiP^SP^-GFP_1–10_ (Figure S3D, bottom). Interestingly, the Fng-GFP_11×7/1–10_ signals in dendrites were in gradation in different stages, with low levels in E3, medium in M3, and high in L3 (Figures S3E and S3F). Taken together, the reconstituted Fng-GFP_11×7/1–10_ signals represent the localization of endogenous Fng at GOPs in C4da dendrites.

### Suppression of dendritic arborization by Fng

**Figure 3.**
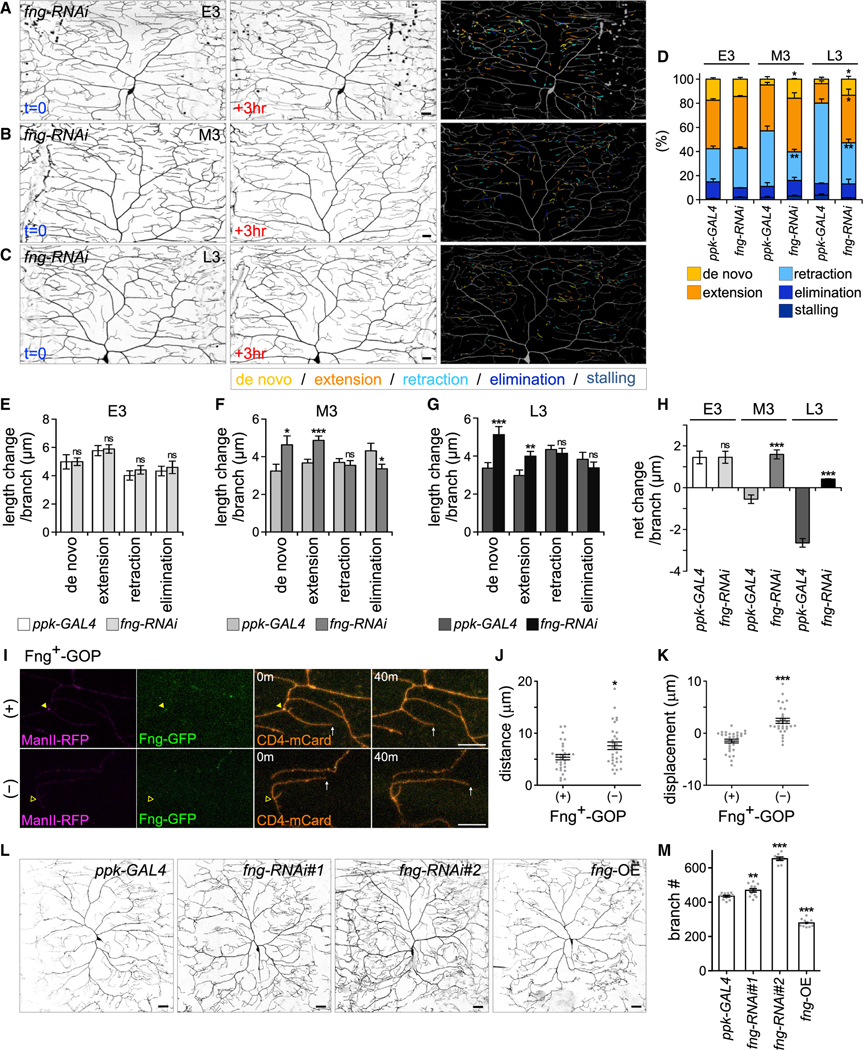
Fng suppresses branch dynamics and dendrite arborization (A–C) Three-hour-span images of *ppk-CD4-tdTom*-labeled C4da neurons with *ppk-GAL4*-driven *fng-RNAi#1* knockdown in the (A) E3, (B) M3, and (C) L3 stages. Changes to branches within the 3 h are color-coded (right). (D) Percentages of branches in different dynamic categories averaged from three *fng-RNAi* neurons in each stage. (E–G) Length change per branch for different dynamic categories in control and *fng-RNAi* neurons in the (E) E3 (n = 466), (F) M3 (n = 608), and (G) L3 (n = 590) stages. (H) Net change per branch for control and *fng-RNAi* neurons in each stage. *ppk-GAL4* control data are from Figures 1D–1F. (I) With *ppk-GAL4*-driven ManII-RFP and Fng-GFP, dynamic branches (white arrows) of CD4-mCard-labeled C4da dendrites were observed in the presence (+) or absence (—) of Fng^+^-GOPs at the dendrite bases (indicated by filled or open yellow arrowheads). (J and K) Dynamics of branches with (n = 30) or without (n = 30) Fng^+^-GOPs show in (J) total moving distance and (K) net displacement of dendritic tips. (L and M) (L) CD4-tdTom-labeled dendrites and (M) total terminal branches of *ppk-GAL4* (n = 12), *ppk-GAL4*-driven *fng-RNAi#1* (n = 12), *fng-RNAi#2* (n = 8), and *fng* overexpression (*fng*-OE, n = 10) C4da neurons. Data and bar graphs represent the mean ± SEM. Compared with control, statistical significance was determined by Student’s t test and shown as *p < 0.05, **p < 0.01, ***p < 0.001, and ns for not significant. Scale bars, 20 μm in (A–C and L) and 10 μm in (I).

Given these concomitant changes in Fng^+^-GOP distribution and terminal branch dynamics in later larval stages, we investigated how Fng loss of function affects dendritic arborization. We compared terminal branch dynamics of *fng-RNAi* knockdown and control C4da neurons in the E3, M3, and L3 stages (Figures 3A–3C). In the E3 stage, the branch dynamics profiles were comparable between control and *fng-RNAi* knockdown (Figures 3D and 3E). However, in the M3 stage, *de novo* and extending branches were increased and branch retraction and elimination were decreased in *fng-RNAi* neurons (Figures 3D and 3F). The overall branch length increased in *fng-RNAi* neurons as opposed to control with length reduction (Figure 3H). In the L3 stage, *de novo* and extending branches were increased, and the retracting branches were significantly decreased in *fng-RNAi* neurons (Figures 3D and 3G), leading to some dendrite growth in the L3 stage (Figure 3H). Taken together, these findings indicate that Fng biases terminal branches from *de novo* formation and extension to retraction and elimination in the M3 and L3 stages.

To examine whether the localization of Fng^+^-GOPs has a more direct effect on branch dynamics, we conducted live imaging for Fng^+^-GOPs and dendritic branches. For comparison, dendritic branches were categorized into Fng^+^-GOP positive or Fng^+^-GOP negative (Figure 3I). We found that Fng^+^-GOP-positive branches were less dynamic, with less moving distance and negative displacement in comparison to Fng^+^-GOP-negative branches, which had a positive averaged displacement (Figures 3J and 3K). The results indicate a correlation between Fng^+^-GOP localization and reduction of branch dynamics and length.

To understand how these changes in branch dynamics ultimately give rise to eventual dendritic arborization patterns, we analyzed total terminal branches in C4da neurons. In the late wandering third-instar larval stage, terminal branch numbers increased in *fng*-knockdown C4da neurons, as shown by two different RNAi lines (Figures 3L and 3M). The dendritic phenotype of *fng-RNAi#2* was stronger, consistent with its stronger knockdown effect (Figure S3G). In contrast, terminal branch numbers were significantly reduced upon *fng* overexpression (Figures 3L and 3M). Thus, Fng functions as a negative regulator suppressing dendritic arborization of C4da neurons.

### Identification of Fur2 as a downregulator of Fng activity in dendritic arborization

**Figure 4.**
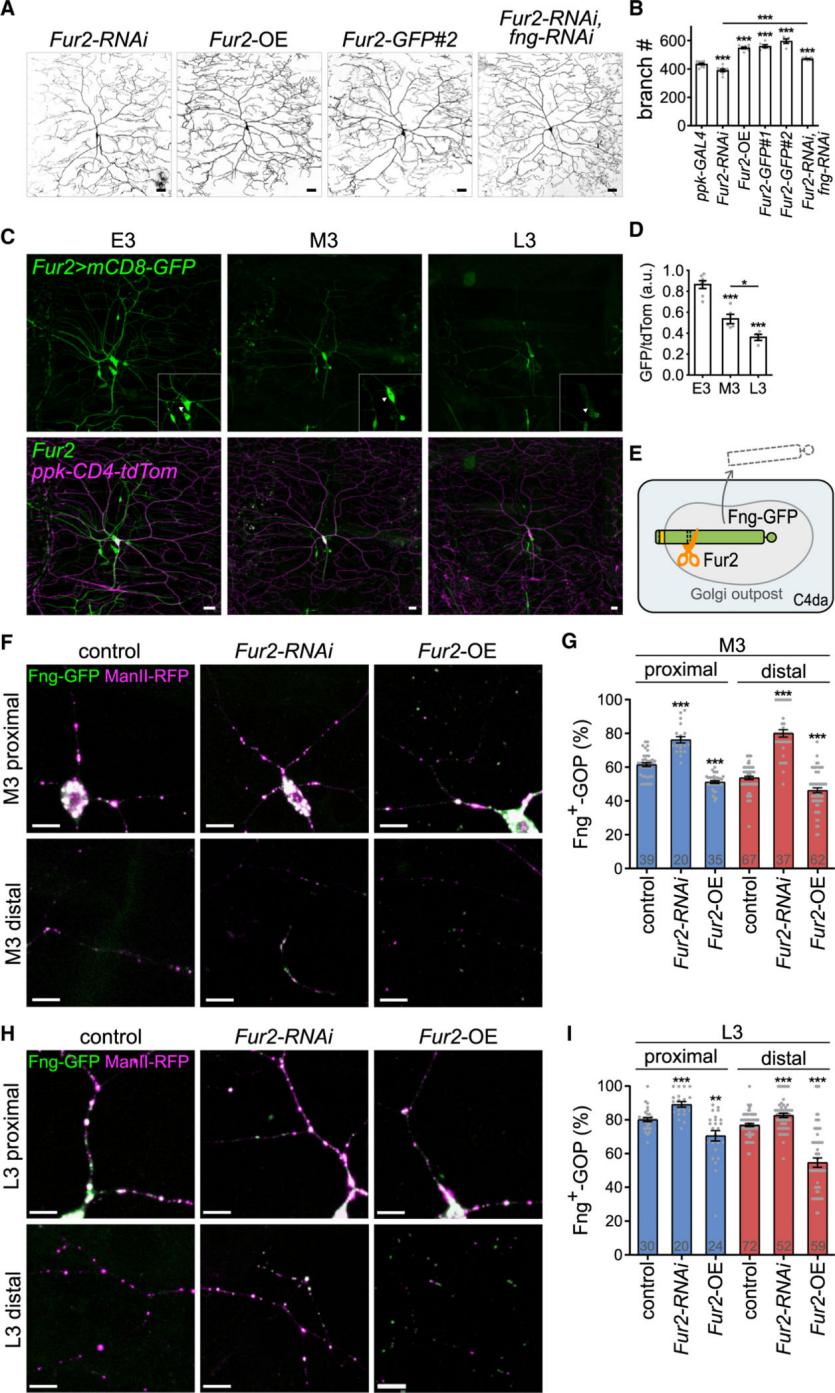
Fur2 promotes branch formation and suppresses Fng^+^-GOPs (A and B) (A) CD4-tdTom-labeled dendrites and (B) total terminal branches of *ppk-GAL4*-driven *Fur2-RNAi* (n = 9), *Fur2* overexpression (*Fur2*-OE; n = 9), *Fur2-GFP#1* (n = 6), *Fur2-GFP#2* (n = 5), and *Fur2-RNAi* and *fng-RNAi* double knockdown (n = 13), relative to *ppk-GAL4* control C4da neurons (from Figure 3M). (C) Expression of mCD8-GFP driven by *Fur2-GAL4* in CD4-tdTom-labeled C4da neurons in the E3, M3, and L3 stages. Insets show enlarged C4da neurons (arrowheads). (D) The mCD8-GFP-to-tdTom intensity ratios within E3 (n = 7), M3 (n = 5), and L3 (n = 4) C4da cell bodies. (E) Schematic shows that Fur2 cleaves Fng-GFP at the Fng luminal domain in the Golgi structures, and truncated Fng without the transmembrane domain is secreted extracellularly. (F and H) Fng-GFP and ManII-RFP were coexpressed by *ppk-GAL4* in control (also coexpressing *lacZ*), *Fur2-RNAi* knockdown, or *Fur2*-overexpressing (*Fur2*-OE) C4da neurons in (F) M3 and (H) L3 stages. (G and I) Fng^+^-GOP percentages in proximal and distal dendrites in (G) M3 and (I) L3 stages. Bar graphs represent the mean ± SEM, and comparisons with control or between groups indicated by lines were processed by Student’s t test with significance shown as *p < 0.05, **p < 0.01, or ***p < 0.001. Scale bars, 20 μm in (A, C) and 10 μm in (F, H).

The increased Fng^+^-GOPs in distal dendrites at later larval stages prompted us to investigate how Fng localization at GOPs is regulated. First, we excluded the regulation of *fng* transcription, as the *fng-GAL4*-driven GFP expression levels were comparable across E3, M3, and L3 stages (Figures S4A and S4B). Fng proteins can be processed by the proprotein convertase subtilisin/kexin (PCSK) (Roebroek et al., 1992, 1995). *Furin 1* (*Fur1*), *Furin 2* (*Fur2*), *amontillado* (*amon*), *Tripeptidyl-peptidase II* (*TppII*), and *Site-1 protease* (*S1P*) encode PCSK proteins in the *Drosophila* genome (Figure S4C), and were tested for C4da dendrite phenotypes by RNAi knockdown. Among the available *PCSK-RNAi* lines, *Fur2*-*RNAi* knockdown reduced the dendritic branch numbers, whereas knockdown of *Fur1*, *amon*, or *TppII* increased them (Figures 4A, 4B, and S4D). Thus, with the contrasting phenotype to the *fng-RNAi* knockdown, we further studied the *Fur2* mutant phenotype by generating *Fur2* mutant MARCM clones for C4da neurons (Lee and Luo, 2001; Matsubara et al., 2011; Shimono et al., 2014). Consistently, the branch numbers of *Fur2*^*A*^ neurons were reduced relative to control (Figures S4E and S4F) and were restored by expressing the *Fur2-GFP* or *Fur2-RFP* transgene, supporting the idea that *Fur2* indeed regulates dendritic arborization (Figures S4E and S4F). Furthermore, *Fur2* overexpression induced excessive branch formation (Figures 4A and 4B). We also examined dendritic arborization in *Fur2-RNAi* and *fng-RNAi* double-knockdown C4da neurons, and the terminal branches were increased compared with *Fur2-RNAi* knockdown (Figures 4A and 4B) and comparable to *fng-RNAi* knockdown (in Figures 3L and 3M, p > 0.05). These comparisons are consistent with the idea that *fng* acts downstream of *Fur2* in dendrite arborization.

To assess *Fur2* expression in C4da neurons, a *GAL4::VP16* knockin allele in the *Fur2* locus was generated (Figure S5A). By driving *UAS-mCD8-GFP* with *Fur2-GAL4*, we detected *Fur2* expression in the nervous system and other tissues of larvae (Figure S5B). Importantly, *Fur2* expression was apparent in peripheral sensory neurons, including C4da neurons (Figure 4C). We found the *Fur2* expression levels in C4da neurons were high in E3, medium in M3, and low in L3 (Figure 4D), pointing to Fur2 as the PCSK candidate responsible for processing Fng in earlier stages.

### Fur2-mediated downregulation of Fng^+^-GOPs in distal dendrites

We continued to study the regulatory role of Fur2 on the distribution of Fng^+^-GOPs in dendrites. First, we found that *ppk-GAL4*-overexpressed Fur2-RFP manifested as discrete puncta in cell bodies and dendrites and, moreover, colocalized with the ManII-GFP signals, indicating that Fur2 could localize at the GOPs (Figure S5C). Fur2-RFP signals also overlapped with Fng-GFP puncta in cell bodies and in the proximal and distal dendrites (Figure S5D). Second, we tested Fur2 regulation of Fng-GFP localization at GOPs. As a type II transmembrane protein (Munro and Freeman, 2000), the Fng N terminus locates in the cytosol and the luminal C-terminal fragment contains putative PCSK processing sites. Processed Fng without membrane anchoring is secreted and inactive (Munro and Freeman, 2000; Shifley and4.9995mm Cole, 2008). Thus, Fur2-processed Fng-GFP would leave GOPs (Figure 4E). Consistently, in *Fur2-RNAi* knockdown C4da neurons, the Fng^+^-GOP percentages were significantly increased in both proximal and distal dendritic regions in the M3 and L3 stages (Figures 4F–4I, S6A, and S6B). In contrast, *Fur2* overexpression caused decreases in the Fng^+^-GOP percentages in proximal and distal dendrites in M3 and L3 stages (Figures 4F–4I, S6A, and S6B). Thus, Fur2 downregulates Fng localization at GOPs.

### Fng is processed by Fur2 at two proteolytic sites

**Figure 5.**
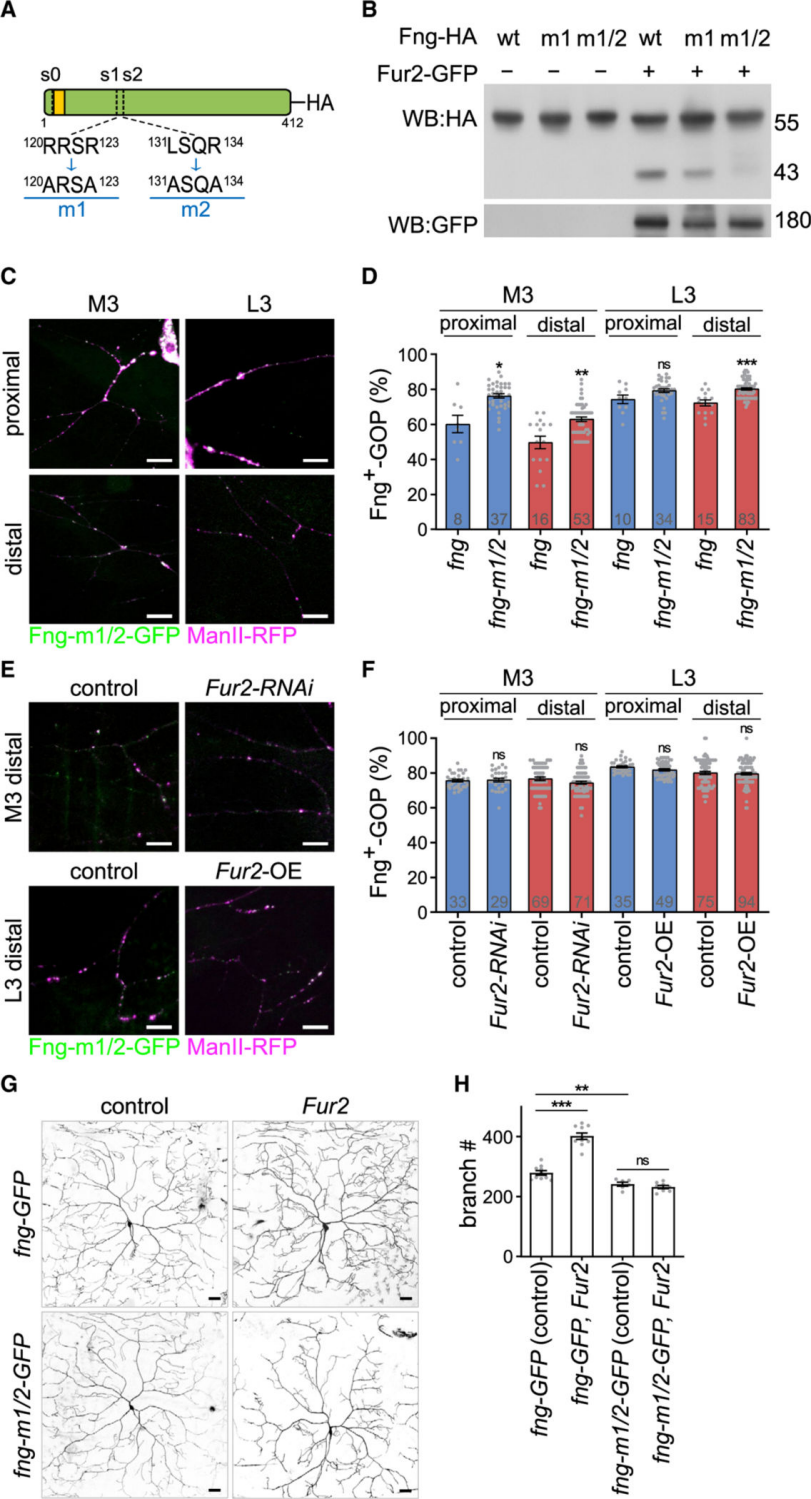
Fur2 regulates Fng through two Golgi-intraluminal processing sites (A) Schematic for Fng protein and its three predicted PCSK cutting sites, s0, s1, and s2. Sequences of the s1 and s2 and the m1 and m2 mutated sites are shown. (B) Western blot showing that S2 cells expressed Fng-HA as a single 55 kDa band (lane 1), but with an extra 43 kDa band upon Fur2-GFP coexpression (lane 4). Upon Fur2-GFP coexpression, the 43 kDa band was reduced for Fng-m1-HA (lane 5) and almost disappeared for Fng-m1/2-HA (lane 6). (C) Proximal and distal dendritic regions of C4da neurons with *ppk-GAL4*-driven Fng-m1/2-GFP and ManII-RFP co-overexpression in the M3 and L3 stages. (D) Fng-m1/2-GFP-localized GOP percentages, compared with those for Fng-GFP (from Figures 3G and 3H). (E) Distal dendritic regions of C4da neurons with *ppk-GAL4*-driven Fng-m1/2-GFP or ManII-RFP, and coexpressing *lacZ* (control), *Fur2-RANi* in M3, or *Fur2*-OE in L3. (F) Fng-m1/2-GFP-localized GOP percentages in proximal and distal dendrites of control, *Fur2-RANi* in M3, and *Fur2*-OE in L3. (G and H) (G) CD4-tdTom-labeled dendrites and (H) total terminal branches of *ppk-GAL4*-driving *fng-GFP* (coexpressing *lacZ* control, n = 10; with *Fur2*, n = 10) and *fng-m1/2-GFP* (*lacZ* control, n = 6; with *Fur2*, n = 8) C4da neurons. Bar graphs represent the mean ± SEM. Comparisons (with Fng in [D], with control in [F], or indicated by lines) were processed by Student’s t test with significance shown as *p < 0.05, **p < 0.01, ***p < 0.001, and ns for not significant. Scale bars, 10 μm in (C, E) and 20 μm in (G).

Next, we determined if Fur2 processes Fng. We identified three probable sites (s0, s1, and s2) within the Fng protein sequence using the ProP 1.0 server (Duckert et al., 2004), fitting the PCSK processing R/LXXR consensus motif (Figures 5A and S5E). The s0 site, located in the cytosol, is unlikely to be processed by Fur2 in the Golgi lumen. We therefore focused on the luminal s1 and s2 sites as targets for Fur2 processing. By replacing the Arg and Lys residues with Ala in the consensus sites, we generated the Fng-m1 single mutant for the s1 site, which is conserved across vertebrate Lunatic fringe proteins (Figure S5E) (Leve et al., 2001), and the Fng-m1/2 double mutant (Figure 5A). S2 cells transfected with *HA*-tagged *fng* transgenes expressed wild-type Fng-HA, Fng-m1-HA, or Fng-m1/2-HA proteins at the expected 55 kDa position (Figure 5B, lanes 1–3). In cotransfection of *Fur2-GFP* and *fng-HA* transgenes, an extra signal representing Fur2-processed product appeared at 43 kDa (lane 4), which was partially reduced for coexpressed Fng-m1-HA (lane 5) and was greatly diminished for coexpressed Fng-m1/2-HA (lane 6). Thus, these results support the idea that Fur2 proteolyzes Fng at the s1 and s2 sites.

To further study the Fur2 processing of Fng in dendrites, we generated a *UAS-fng-m1/2-GFP* transgene. As observed for Fng-GFP, Fng-m1/2-GFP also localized to ManII-RFP-positive GOPs (Figures 5C and S6C). Interestingly, while Fng-m1/2-GFP displayed a slightly higher total number of puncta per neuron (204 ± 7.6) than Fng-GFP (189 ± 17.8), without statistical significance, Fng-m1/2-GFP presented a higher percentage of GOP colocalization than Fng-GFP in distal dendritic regions in the M3 and L3 stages (Figure 5D). To test whether the proteolytic sitemutated Fng-m1/2 is resistant to Fur2 processing, we performed *Fur2-RNAi* knockdown in Fng-m1/2-GFP-expressing neurons in M3, when *Fur2* expression is high (Figure 4C). We found that *Fur2-RNAi* knockdown did not increase the Fng-m1/2-GFP-localized GOP percentages in either proximal or distal regions (Figures 5E, 5F, and S6D). Likewise, *Fur2* overexpression in C4da neurons in L3, when *Fur2* expression is low (Figure 4C), had no effect on Fng-m1/2-GFP-localized GOP percentages (Figures 5E, 5F, and S6D). In contrast to the sensitivity of GOP-localized Fng-GFP to Fur2 (Figures 4F–4I), the insensitivity of Fng-m1/2 indicates that Fur2 processes Fng at the s1 and s2 sites.

Next, we examined if Fur2 downregulates Fng activity to regulate dendritic arborization. Overexpression of *fng* reduced dendritic branches (Figures 3L and 3M), which was suppressed by the overexpression of *Fur2*, leading to an increase in the branch number (Figures 5G and 5H). Overexpression of *fng-m1/2* also strongly suppressed dendritic arborization, resulting in fewer dendritic branches than *fng* overexpression (Figures 5G and 5H). Importantly, the suppressed dendritic arborization caused by *fng-m1/2* overexpression was not ameliorated by *Fur2* co-overexpression (Figures 5G and 5H). Based on these findings, we propose that Fur2 inhibits Fng activity by proteolytically processing Fng at the s1 and s2 sites to promote dendrite growth.

### Non-canonical Notch signaling mediates Fng activity in dendritic branch retraction

**Figure 6.**
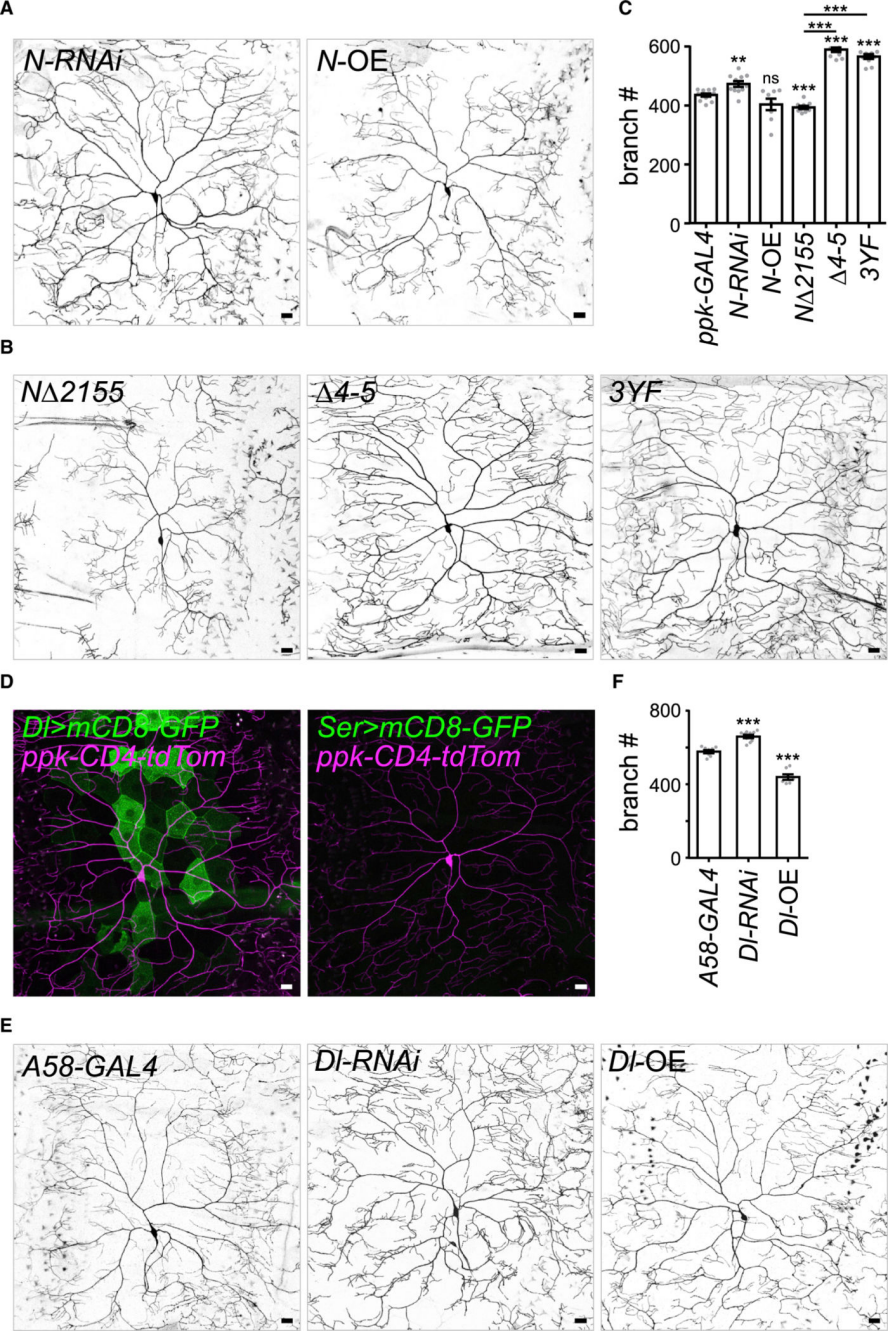
Non-canonical N signaling suppresses dendritic arborization (A–C) (A and B) CD4-tdTom-labled dendrites and (C) total terminal branches of *ppk-GAL4-*driven *N-RNAi* (n = 11), *N* overexpression (*N*-OE; n = 8), *N*Δ*2155* (n = 10), *N*Δ*2155(*Δ*4–5)* (n = 10), and *N*D*2155(3YF)* (n = 10), relative to *ppk-GAL4* control C4da neurons (from Figure 3M). (D) *Dl-GAL4-* and *Ser-GAL4-*driven mCD8-GFP and *ppk-CD4-tdTom*-labeled C4da neurons of L3-stage larvae. (E and F) (E) CD4-tdTom-labeled dendrites and (F) total terminal branches of epidermal *A58-GAL4*-driven *Dl-RNAi* (n = 10) and *Dl* overexpression (*Dl*-OE; n = 7) compared with *A58-GAL4* control (n = 8) C4da neurons. Bar graphs represent the mean ± SEM, and comparisons with control or between groups indicated by lines were processed by Student’s t test with significance shown as **p < 0.01, ***p < 0.001, and ns for not significant. Scale bars, 20 μm.

Fng transfers GlcNAc to O-linked fucose on the extracellular epidermal growth factor repeats of the N receptor (LeBon et al., 2014; Moloney et al., 2000). Accordingly, we investigated whether N also regulates C4da dendritic branching. Total dendritic branch numbers of C4da neurons were significantly increased by *ppk-GAL4*-driven *N-RNAi* (Figures 6A and 6C). When *N* was overexpressed in C4da neurons, the reduction of branch numbers was not statistically significant (Figures 6A and 6C). However, dendritic complexity and total dendritic length were both significantly reduced (Figures S7A and S7B). Thus, N may function in C4da neurons to suppress dendrite growth.

Activation of the canonical N signaling pathway requires the DNA-binding factor Su(H) for gene expression (Bray, 2006). However, we did not detect expression of *Su(H)GBE-GAL4* (Zeng et al., 2010) in C4da neurons (Figure S7C). Therefore, we explored whether non-canonical N signaling is active in dendritic branching, as per its role in axonal patterning (Le Gall et al., 2008). C-terminal-truncated NΔ2155, in which two of the three Su(H)-binding sites have been removed, preferentially functions in the non-canonical pathway (Kannan et al., 2017, 2018). Overexpression of NΔ2155 in C4da neurons caused a significant reduction in branch numbers, and the effect was stronger and more robust than for overexpression of full-length N (Figures 6B and 6C). Non-canonical N activity is mediated partly through Disabled (Dab), which binds to the Ram A domain of N (Giniger, 1998). Deletion of the Dab-binding site in NΔ2155 (NΔ2155(Δ4–5)) abolished the ability of NΔ2155 to suppress dendritic branching (Figures 6B and 6C). We obtained a similar result upon overexpression of NΔ2155(3YF), in which the three Tyr residues required for N regulation of the Abelson tyrosine kinase pathway have been mutated to Phe (Figures 6B and 6C). Indeed, overexpression of NΔ2155(Δ4–5) or NΔ2155(3YF) caused branch increases, as opposed to NΔ2155 overexpression. Together, these results strongly support the idea that non-canonical N signaling inhibits dendritic branching.

Fng-mediated modification of the N receptor enhances interaction between N and the Delta (Dl) ligand and suppresses its - interaction with the Serrate (Ser) ligand (Fleming et al., 1997; Klein and Arias, 1998). Therefore, we examined expression patterns of *GAL4*-trapped *Dl*^*05151-G*^ (*Dl-GAL4*) and *Ser-GAL4*. Interestingly, *Dl-GAL4* expression was detected in epidermal cells, but lacked apparent signals in C4da neurons (Figure 6D). Moreover, we could not detect expression of *Ser-GAL4* in C4da neurons or epidermal cells (Figure 6D). Meanwhile, *Dl-RNAi* knockdown in epidermal cells caused an increase in dendritic branch number, whereas *Dl* overexpression significantly impaired dendrite growth (Figures 6E and 6F). Taking these results together, we propose that Dl expressed in epidermal cells activates non-canonical N signaling in C4da neurons to suppress dendritic arborization.

### Fng downregulates actin dynamics in dendritic branches

**Figure 7.**
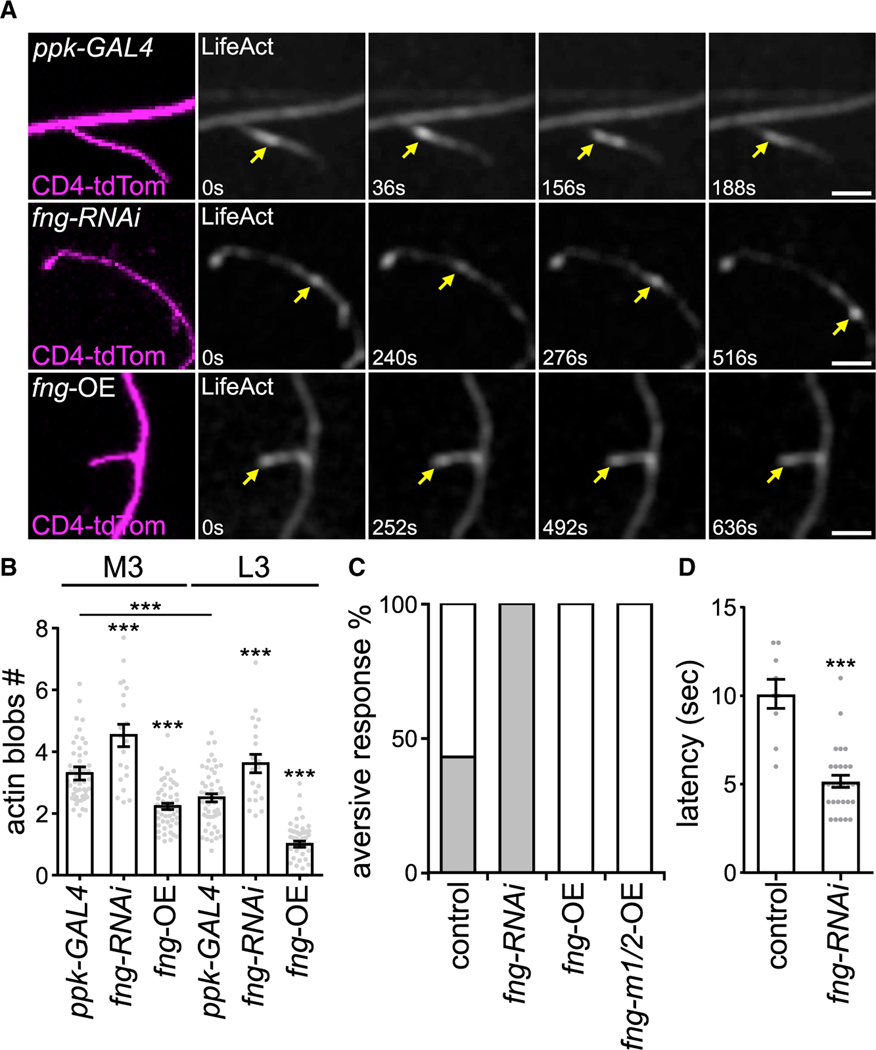
Fng suppresses F-actin dynamics in dendrites and thermal nociceptive responses of larvae (A and B) (A) Actin blobs (yellow arrows) were labeled by *ppk-GAL4*-driven LifeAct-GFP in CD4-tdTom-labeled C4da neurons, and (B) the moving actin blob numbers (in 10 μm segments within 10 min) were quantified, in *ppk-GAL4* control (n = 50), *fng-RNAi* (n = 20), and *fng*-overexpressing (*fng-*OE; n = 50) C4da neurons in the M3 or L3 stage. (C) Percentages (gray) of larvae displaying rolling and escape behavior induced by thermal stimulation in *ppk-GAL4*-driven *lacZ* control (n = 21), *fng-RNAi* (n = 29), *fng*-OE (n = 11), and *fng-m1/2*-OE (n = 7) C4da neurons. (D) Response latency for control (n = 9) and *fng-RNAi* (n = 29). Bar graphs represent the mean ± SEM, and comparisons with control or between data groups indicated by lines were processed by Student’s t test with significance shown as ***p < 0.001. Scale bars, 5 μm.

Given that non-canonical N signaling promotes actin polymerization and branching of actin structures (Tremmel et al., 2013; Wesley et al., 2011), we investigated whether Fng modulates actin dynamics in dendrites. Actin blobs are dynamic entities in dendrites that promote branching, while their retarded dynamics compromise dendritic growth (Nithianandam and Chien, 2018). Accordingly, we assayed actin blob dynamics by monitoring F-actin-binding LifeAct-GFP signals in C4da dendrites (Figure 7A). From M3 to L3, the numbers of moving actin blobs declined, similar to the previous study (Nithianandam and Chien, 2018). Interestingly, *fng-RNAi* knockdown resulted in increased numbers of dynamic actin blobs in both M3 and L3 stages, whereas *fng* overexpression significantly reduced the numbers of dynamic actin blobs in both stages (Figure 7B). Thus, the population of dynamic actin blobs in dendrites is negatively regulated by Fng in C4da neurons, which correlates with terminal branch dynamics in these two stages.

### Responses to thermal stimulation are correlated with dendritic branch numbers

C4da neurons are nociceptors, perceiving noxious thermal stimuli and inducing escape behaviors, including withdrawal and rolling of *Drosophila* larvae (Chattopadhyay et al., 2012; Tracey et al., 2003). With the role of *fng* in regulating C4da dendritic branching identified, we tested whether it also modulates nociceptive thermal responses. With heat stimulation at 42°C, ~40% of control larvae responded to the tests, whereas *fng-RNAi* knockdown larvae displayed 100% responsiveness (Figure 7C). In contrast, larvae with overexpressed *fng* or *fng-m1/2* in C4da neurons showed no responsiveness to nociceptive stimulation (Figure 7C). The latency in response to stimulation showed that *fng-RNAi* knockdown larvae presented a shorter response time compared with control (Figure 7D). Thus, our results indicate that Fng in C4da neurons suppresses larval responses to nociceptive heat stimulation in L3.

## DISCUSSION

In this study, we identified an unappreciated GOP function in dendritic arborization: a subset of Fng-positive GOPs that are proposed to function in branch retraction. Our genetic analyses suggest that Fng downregulates actin dynamics to promote branch retraction. The Fng-modified N receptor is activated in *trans* by the Dl ligand from epidermal cells to transduce the non-canonical pathway. Moreover, we demonstrate that Fng is proteolyzed by the proprotein convertase Fur2 for temporal regulation. We propose that GOPs serve as a venue for the convergence of spatiotemporal signals to regulate Fng activity and shape dendritic arborization.

### GOP heterogeneity confers functional diversity on dendrite dynamics

Golgi fragments carrying different sets of molecules are proposed to constitute distinct functional units in cells. In mammalian cells, proteins with relevant functions are compartmentalized into subdomains of the Golgi apparatus (Chen et al., 2017). Discrete Golgi units harbor various enzymes dedicated to specific signaling pathways in *Drosophila* wing-disc cells (Yano et al., 2005). In this study, we also show distinctive localization of Fng and Rho at somatic Golgi structures and dendritic GOPs (Figure S2). Thus, heterogeneous GOPs host different proteins, enabling them to exert versatile functions in neurons. In C4da neurons; anterograde GOP movement precedes branch extension, whereas branch retraction follows retrograde GOP movement (Ye et al., 2007). The GOP dynamics mainly account for the growing phase of dendrites. Instead, Fng-localized GOPs promote retraction of terminal branches in the L3 stage. Accordingly, alterations in GOP composition enable a shift in branch dynamics, ultimately shaping overall dendrite morphology. GOPs in neurons may be similar across different species. For instance, GM130 is required for GOP biogenesis in both *Drosophila* and mammalian neurons (Horton et al., 2005; Zhou et al., 2014). Mammalian GOPs and ER-Golgi intermediate complex structures are also identified in lower- and higher-order dendrites (Horton et al., 2005; Mikhaylova et al., 2016; Quassollo et al., 2015). Given the conservation of Fng and N proteins among mammals (Cohen et al., 1997; Johnston et al., 1997), the functions of Fng-localized GOPs may also be conserved.

### Fur2 regulates Fng localization at GOPs and Fng activity

Fur2 expression declines gradually from the E3 to the L3 stage, correlating with a switch from dendritic branch extension to retraction. Fur2 could proteolyze Fng and lessen Fng^+^-GOP numbers in dendrites in E3. Lowered Fur2 levels in the M3 and L3 stages allow unprocessed Fng^+^-GOPs to be transported to distal dendrites. Proteolytic processing of Fng proteins represents an efficient way to control their activity. In *Drosophila* wing-disc cells, Fng tethered to a constitutive Golgi-retaining peptide is inert to processing, exhibiting a gain-of-function phenotype, while Fng tethered to a secretory signal manifests as a loss-of-function mutant (Munro and Freeman, 2000). In this study, the Fng-GFP protein would be expelled from GOPs when processed by Fur2, leading to its inactivation. Instead, Fng-m1/2-GFP located at GOPs is resistant to Fur2 processing and functions constitutively to suppress dendrite arborization (Figure 5). PCSK5, the closest mammalian homolog of Fur2, is expressed in nervous systems and functions to process mammalian Fng proteins, implying a possible conserved mechanism in dendrite arborization (Essalmani et al., 2008; Roebroek et al., 1992; Uhlé n et al., 2015).

### Spatial and temporal regulatory mechanisms converge onto non-conventional N signaling in dendrites

N signaling regulates dendritic branching in mammalian neurons at several levels, varying according to neuronal type and differentiation stage (Breunig et al., 2007; Ding et al., 2016; Muroyama et al., 2016; Sěstan et al., 1999). Activation of N signaling through spatial or temporal cues could determine how N signaling participates in shaping neuronal morphology. O-glycosylation of N by Fng biases its interaction toward the ligand Dl than toward Ser (Brückner et al., 2000; Fleming et al., 1997; LeBon et al., 2014). Concomitantly, Dl is expressed in epidermal cells, providing the spatial cue for activating N signaling to promote terminal branch retraction. We excluded the involvement of conventional N signaling mediated by the nuclear factor Su(H) (Figure S7C), which may instead be involved in dendritic patterning at earlier stages of C4da neuronal development (Wang et al., 2020). Our data suggest that non-canonical N signaling promotes branch retraction by regulating actin dynamics, likely via downstream effectors to modulate polymerization and branching of actin filaments, similar to regulating axonal extension in *Drosophila* (Kannan et al., 2018; Kuzina et al., 2011). In photoreceptors, the actin regulator Enabled (Ena), the downstream effector of non-canonical N signaling, localizes at somatic Golgi complexes and modulates Golgi structures, likely through actin remodeling (Kannan et al., 2014). Thus, activation of non-canonical N signaling could also impede local actin dynamics to regulate GOP distribution in dendrites.

### Physiological relevance of dendritic pruning in late third-instar larvae

In L3, the pruning process mainly manifests as retraction and elimination of terminal branches, which is quite distinct from the bulk dendritic pruning during the pupal stage. Some genotypes in our study showed less dramatic terminal phenotypes, as expected, which could be due to other mechanisms that compensate for the defect at the wandering stage (Nithianandam and Chien, 2018). However, it remains puzzling why C4da dendrites undergo branch retraction in the L3 stage prior to the large-scale pruning in the pupal stage. As late L3 larvae advance to the wandering stage, they crawl out from food substrates to drier places for pupariation (Brown et al., 2017). C4da neurons are nociceptors for strong and harmful stimulations such as heat and UV light (Gorczyca et al., 2014; Xiang et al., 2010). A reduction in dendritic branches in the L3 stage may desensitize C4da neurons, rendering L3-staged larvae more adaptable to stronger light and other nociceptive stimuli from their surroundings in the absence of shelter. The mechanistic aspect of the pruning process described herein could underpin developmental pruning, physiological plasticity, and human disorders such as chronic stress and atopic dermatitis (Lupien et al., 2018; Takahashi et al., 2019).

### Limitations of the study

First, while we showed the correlation of Fng^+^-GOP localization with branch retraction, whether it also correlates with actin dynamics in dendrites awaits further study. This limitation is 2-fold: (1) four different fluorescent proteins are required to tag Fng, GOP, actin, and dendrites for live imaging in living larvae and (2) the dynamics of actin movement (in seconds) and branch changes (in tens of seconds) are quite different, and the “local” range of correlation could be difficult to gauge. Second, considering the diversified dendritic trees in different types of neurons, it would be interesting to explore the developmental and cellular contexts for employing the Fur2-Fng-N axis in dendrite arborization.

## STAR★METHODS

### RESOURCE AVAILABILITY

#### Lead contact

Further information and requests for resources and reagents should be directed to and will be fulfilled by the lead contact, Cheng-Ting Chien (ctchien@gate.sinica.edu.tw).

#### Materials availability

Fly lines and plasmids generated in this study are available upon request.

#### Data and code availability

- All data reported in this paper will be shared by the lead contact upon request.
- This paper does not report original code.
- Any additional information required to reanalyze the data reported in this paper is available from the lead contact upon request.

### EXPERIMENTAL MODEL AND SUBJECT DETAILS

#### Experimental animals

All flies were reared on normal cornmeal food and in incubator at 25°C with 12 h-light/12 h-dark cycle. Fly larvae of both sexes were used in all experiments. For larval staging experiments, embryos were collected from grape agar plates, and larvae hatched within 6 h were collected and analyzed at three sub-stages, i.e., early third-instar (E3) at 48–54 h after larva hatching (ALH), mid third-instar (M3) at 66–72 h ALH, and late third-instar (L3) at 84–90 h ALH. Wandering third-instar larvae were used for analyzing terminal dendrite phenotypes. Fly lines used in this study were listed in the Key Resources Table.

#### Cell lines

*Drosophila* S2 cells were cultured at 25°C in Schneider’s *Drosophila* medium (21720024, Thermo Fisher Scientific) supplemented with 10% fetal bovine serum (12676029, Thermo Fisher Scientific), and 1% penicillin/streptomycin (15140122, Thermo Fisher Scientific).

### METHOD DETAILS

#### Generation of transgenes and transgenic flies

Full-length cDNAs of *fng*, *rho*, and *sfl* were amplified by polymerase chain reaction (PCR) from respective plasmids (Yano et al., 2005) to generate *UAS-fng-GFP*, *UAS-sfl-GFP*, and *UAS-GFP-rho*. Site-directed mutagenesis of *fng* was performed by PCR to generate *fng-m1* (R120A and R123A) and *fng-m1/2* (R120A, R123A, L131A, and R134A). *Fur2* cDNA was amplified by PCR from the *UAS-Fur2* fly. These cDNAs were subcloned to generate *UAS-fng-m1/2-GFP, UAS-Fur2-GFP#1*, *UAS-Fur2-GFP#2*, *UAS-fng-HA*, *UAS-fng-m1-HA*, *UAS-fng-m1/2-HA*, and *UAS-Fur2-RFP* plasmids. *fng*^*N*^ (cDNA for Fng amino acids 1–48) and *BiP*^*SP*^ (cDNA from BiP amino acids 1–18) (Yamaji et al., 2008) were fused with *GFP*_*1–10*_ (70,219, Addgene) to generate *UAS-fng*^*N*^*-GFP*_*1–10*_ and *UAS-BiP*^*SP*^*-GFP*_*1–10*_. *mCard* was amplified from *UAS-AV-mCard* (Sapar et al., 2018) for generating *ppk-CD4-mCard*. These plasmids were used to generate transgenic flies by embryo microinjection or for S2 cell transfection assays.

*fng-GFP*_*11×7*_ (seven repeats of split GFP_11_ fused and knock-in to the C-terminus of *fng* with SR linker) and *Fur2-GAL4* (*GAL4::VP16* knock-in at the *Fur2* locus) were generated through CRISPR/Cas9-mediated genome editing with homology-dependent repair (HDR), handled by WellGenetics Inc. (Taiwan).

#### Microscopy and image analysis

Live imaging of C4da dendrites was conducted as previously described (Lin et al., 2015; Yang et al., 2011) with modification. Living larvae were mounted on a coverslip (22 mm, Superior Paul Marienfeld) in a cavity created by using double-sided tape (3M^™^ Removable Repositionable Tape 665, 3M), and the dorsal side of larvae was covered with a round coverslip (40 mm coverslips, Bioptechs) for imaging in a customized imaging chamber. Dendritic arbors of the dorsal CD4-tdTom-labled (Han et al., 2011) C4da neurons in the A5-A7 segments were imaged through 20× (NA 0.8, Plan-Apochromat 20×/0.8, Carl Zeiss) objective lens on LSM710 confocal microscope (Carl Zeiss) or spinning disk confocal microscope CSU-X1 (Carl Zeiss). After image acquisition, larvae were released and kept on grape juice agar plates in a 25°C incubator for 3 h, before reimaging the same C4da neurons. Image data for larvae undergoing the entire experimental process were further quantified for branch length changes within the 3-h time-period. Stalled branches were branches with changes in length <0.2 μm. Length changes from terminal branches were summed and averaged to show net change per branch.

Live-imaging subcellular distribution of fluorescence-tagged proteins in C4da dendrites were performed in larvae prepared as described above. Filter paper soaked with desflurane (Suprane, Baxter Co.) was placed into the chamber for 2 min to sedate the larvae for imaging. Images were taken through 40× (NA 1.2, C-Apochromat 40×/1.2 W Corr M27) objective lens with Immersol W (444969, Carl Zeiss), or 63× (NA 1.4, C Plan-Apochromat 63×/1.4 Corr M27) objective lens with Immersol^™^ 518F (444960, Carl Zeiss) on LSM710 or LSM880 confocal microscope (Carl Zeiss). Fluorescent puncta with colocalization of Fng-GFP and ManII-RFP signals were counted as Fng-GFP positive GOPs (Fng^+^-GOPs), and the Fng^+^-GOP percentage was fraction of Fng^+^-GOPs among all GOPs. For quantification of Fng^+^-GOP percentages, the proximal dendritic regions were defined as within 50 μm dendritic segments from the soma, and the distal dendritic regions were defined as within 50 μm segments from the branch tips.

Live imaging of C4da dendritic branches with or without Fng^+^-GOPs located at the branching sites were conducted in sedated larvae prepared as described above, with image acquisition at every 5 min in 40 min. Fng^+^-GOP-positive branches were defined with the averages of both Fng-GFP and ManII-RFP signal intensities higher than background intensities plus 4-fold of standard deviation. If the differences between signal intensity and background were less than 2.5-fold standard deviation, the branches were defined as Fng^+^-GOP-negative. Images were acquired through 40× objective lens with Immersol W on LSM900 confocal microscope (Carl Zeiss). Moving distances and displacements of dendritic branch tips were quantified in 40-min periods.

To quantify C4da dendritic branch numbers, larvae were gently crushed to remove internal organs and mounted in medium of 90% glycerol plus 2.5% DABCO (290734, Sigma-Aldrich) with coverslips for imaging. Images were acquired through 20× objective lens on LSM710 confocal microscope. Total dendritic lengths were analyzed by Imaris (Bitplan Inc.) through ‘Filament Tracer’ function (with manual curation).

To compare expression levels of *GAL4-*driven GFP in C4da neurons, background intensities (signals outside C4da neurons) of respective fluorescent channels were subtracted from the fluorescence intensities of the reporters GFP or tdTom within the somatic region, and the GFP/tdTom ratios were analyzed and shown as arbitrary units (a.u.). Female larvae were dissected and imaged for revealing *Fur2-GAL4-*driven GFP expression patterns.

Live imaging and analysis of dynamic actin blobs in C4da dendrites was achieved by following the method described previously (Nithianandam and Chien, 2018). Fluorescent LifeAct-GFP in C4da dendrites was imaged in living and non-anesthetized larvae, with consecutive image stacks of actin blobs being taken at 0.2 Hz for 10 min through 40× objective lens on LSM880. After quantifying the numbers of actin blobs, data distributions were checked by Kolmogorov-Smirnov normality test, while outliers were excluded with the ROUT method. Fluorescent signals from LifeAct-GFP in the z-axis were summed using the ‘Maximum Intensity Projection’ function (Zen 2012) and ‘Gaussian function 3’ of MetaMorph (Molecular Devices) to detect actin blobs.

#### Analysis of ManII-GFP or Fng-GFP puncta distributions

ManII-GFP or Fng-GFP and tdTom-labeled dendritic arbors were imaged simultaneously for analysis. The C4da dendrites were manually tracked using a graphics tablet (Sapphire CTE-431, Wacom) and Adobe Photoshop (CS6) to generate camera lucida of dendritic arbors. Fluorescence signals of ManII-GFP and Fng-GFP puncta within dendritic segments were analyzed by the ‘Analyze Particles’ function in ImageJ (version 1.50, NIH) that define the centroids of each punctum with the parameters 0.0–1.0 for ‘circularity’ and 5–100 pixels for ‘particle size’. Positions of puncta were recorded and superimposed on the coordination plane, with the somatic centroid as the origin. To quantify the distributions of puncta, dendritic fields were divided into concentric regions with 20 μm interval steps, and numbers of puncta in each region were counted. To normalize the distributions against different dendritic arbor sizes, the mean radii of dendritic fields were determined for the respective larval stages. The square root of the convex polygon area covering the dendritic field divided by pi was taken as representative of dendritic field radius, and the mean radius was quantified for each larval stage and used for normalization, shown as the normalized radius (n.r.) from the soma (0.0) to the mean radius (1.0), or beyond (>1.0). The median radii of puncta accumulative distributions or normalized distributions were also analyzed.

#### Immunohistochemistry

Larvae in the E3, M3 or L3 stage were dissected in cold PBS and fixed in 4% paraformaldehyde (15170, Electron Microscopy Sciences) with 0.2% Triton X-100 (T8787, Sigma-Aldrich) with PBS, and were blocked with 5% normal donkey serum in PBST. The larval fillets were incubated with primary antibodies, chicken anti-GFP (1:500) and rabbit anti-GM130 (1:500) at 4°C overnight, washed and then incubated with secondary antibodies, anti-chicken 647 (1:500) and anti-rabbit Cy3 (1:500), for one hour. Wing discs of L3 larvae were dissected and underwent immunostaining for GM130. After immunostaining, samples were mounted in 87% glycerol and imaged through 20× or 63× objective lens with Immersol^™^ 518F on LSM880.

#### Western blot

Western blot was conducted as previously described (Lin et al., 2015). S2 cells of 2×10^6^ were cultured in 6-well dishes and transfected with *Trans*IT^®^-Insect Transfection Reagent (MIR 6100, Mirus) for the plasmids *pAc-GAL4* and *pUAS-fng-HA, pUAS-fng-m1-HA, pUAS-fng-m1/m2-HA,* or *pUAS-Fur2-GFP*. After 72 h, transfected cells were subjected to 1,500 G centrifugation at 4°C for 5 min and lysed with mRIPA buffer [1% Nonidet P-40, 0.5% Triton, 50 mM Tris-HCl (pH 7.5), 150 mM NaCl, 1 mM EDTA], and protease inhibitor cocktail (cOmplete^™^, 11836145001, Roche). The lysates were centrifuged for 30 min at 4°C, and the supernatant was added 5 μL of 5X SDS loading buffer (250 mM Tris pH 6.8, 25% glycerol, 10% SDS, 1% β—mercaptoethanol, 0.1% bromophenol blue). Protein concentrations in supernatants were determined using a BioDrop DUO+ spectrophotometer (80–3006-68, Biochrom). Proteins were loaded in 8% protein gels for electrophoresis and transferred to nitrocellulose membrane (BioRad) for immunoblotting. The primary antibodies used for Western blotting were rabbit anti-GFP (1:5000) and rabbit anti-HA (1:5000). HRP conjugated secondary antibodies (1:5,000) were diluted in TBS/0.1% Tween-20/5% BSA. The membranes were incubated with primary antibodies rocking at 4°C overnight, washed 3 times for 15 mins with TBS, probed with secondary antibody for 1 h at room temperature, and washed 3 times for 10 min. Chemiluminescence processing was conducted according to the manufacturer instructions (NEL105001EA, Western Lightning^™^ Plus-ECL, PerkinElmer) with an OPTIMAX (Protec) film processor.

#### Nociceptive heat response assay

Nociception behavior assay was performed as previously described (Chattopadhyay et al., 2012; Tracey et al., 2003). We built a thermal probe with a metal tip (~0.1 mm^2^) mounted in an aluminum heater block within which a sensor is embedded for temperature feedback to the controller. We applied 42°C to induce larval withdraw responses in 20 s, which were video recorded and analyzed for response and latency.

### QUANTIFICATION AND STATISTICAL ANALYSIS

Statistical analyses were processed with Prism (6.0, Graphpad). Comparisons between two groups of data were analyzed by two-tailed unpaired Student’s t test. For more than two groups of data, two-way ANOVA and subsequent Bonferroni post-hoc analysis were used. Statistical significance were annotated as * p value < 0.05, ** p value < 0.01, *** p value < 0.001. Sample size for each experiment is denoted in the Table S1. Statistical analyses and plots generation were done by Excel (Microsoft) and Prism.

## Supplementary Material

1

2

## Figures and Tables

**Table:** KEY RESOURCES TABLE

REAGENT or RESOURCE	SOURCE	IDENTIFIER
Antibodies		

Anti-GFP (chicken)	Abcam	Cat#13970; RRID:AB_300798
GM130 antibody - *Drosophila* Golgi/Cis-Golgi Marker	Abcam	Cat#30637; RRID:AB_732675
anti-GFP (rabbit)	Thermo Fisher Scientific	Cat#A-11122; RRID:AB_221569
anti-HA (rabbit)	GeneTEX	Cat#GTX115044; RRID:AB_10622369
Alexa Fluor 647-AffiniPure Donkey Anti-Chicken IgY (IgG) (H + L)	Jackson ImmunoResearch Labs	Cat#703-605-155; RRID:AB_2340379
Cy3-AffiniPure F(ab’)2 Fragment Donkey Anti-Rabbit IgG (H + L)	Jackson ImmunoResearch Labs	Cat#711-166-152; RRID:AB_2313568
Peroxidase-AffiniPure Goat anti-rabbit IgG	Jackson ImmunoResearch Labs	Cat#111-035-003; RRID:AB_2313567

Experimental models: Cell lines		

*Drosophila* S2 cells		RRID:CVCL_Z232

Experimental models: Organisms/strains		

*ppk-GAL4(II)*	BDSC	RRID:BDSC_32078
*ppk-GAL4(III)*	BDSC	RRID:BDSC_32079
*ppk-CD4-tdTomato(II)*	BDSC	RRID:BDSC_35844
*ppk-CD4-tdTomato(III)*	BDSC	RRID:BDSC_35845
*UAS-mCD8GFP*	BDSC	RRID:BDSC_32184
*UAS-mCD8RFP*	BDSC	RRID:BDSC_32218
*UAS-ManII-GFP*	BDSC	RRID:BDSC_65248
*UAS-ManII-tagRFP*	BDSC	RRID:BDSC_65249
*FRT19A*	BDSC	RRID:BDSC_1744
*Fur2A*	BDSC	RRID:BDSC_57092
*fngGawB*	BDSC	RRID:BDSC_9891
*UAS-fng*	BDSC	RRID:BDSC_8553
*UAS-N*	BDSC	RRID:BDSC_52309
*UAS-Dl-GFP*	BDSC	RRID:BDSC_8610
*UAS-Fur2*	BDSC	RRID:BDSC_63081
*UAS-lacZ*	BDSC	RRID:BDSC_3955
*UAS-fng-RANi#1*	BDSC	RRID:BDSC_25947
*UAS-Fur1-RNAi*	BDSC	RRID:BDSC_42481
*UAS-Fur2-RNAi*	BDSC	RRID:BDSC_42577
*UAS-TppII-RNAi#1*	BDSC	RRID:BDSC_65178
*UAS-amon-RNAi#1*	BDSC	RRID:BDSC_41635
*UAS-amon-RNAi#2*	BDSC	RRID:BDSC_44001
*UAS-N-RNAi*	BDSC	RRID:BDSC_35640
*Dl-GAL4*	BDSC	RRID:BDSC_67047
*Ser-GAL4*	BDSC	RRID:BDSC_6791
*UAS-LifeAct-GFP*	BDSC	RRID:BDSC_35544
*UAS-fng-RNAi#2*	VDRC	RRID:BDSC_51977
*UAS-Dl-RNAi*	VDRC	v3720; RRID:FlyBase_FBst0461889
*UAS-TppII-RNAi#2*	VDRC	v26348; RRID:FlyBase_FBst0456368
*hs-FLP, tub-GAL80, FRT19A; 109(2)80Gawb, UAS-mCD8-GFP, SOP-FLP*	DGGR	RRID:DGGR_109946
*UAS-CFP-Golgi*	(Satoh et al., 2005)	N/A
*A58-GAL4*	(Galko and Krasnow, 2004)	N/A
*UAS-NΔ2155*	(Kannan et al., 2018)	N/A
*UAS-NΔ2155(Δ4–5)*	(Kannan et al., 2018)	N/A
*UAS-NΔ2155(3YF)*	(Kannan et al., 2018)	N/A
*UAS-fng-GFP*	This study	N/A
*UAS-GFP-rho*	This study	N/A
*UAS-sfl-GFP*	This study	N/A
*fng-GFP11×7*	This study	N/A
*UAS-fngN-GFP1-10*	This study	N/A
*UAS-BiPSP-GFP1-10*	This study	N/A
*ppk-CD4-mCard*	This study	N/A
*Fur2-GAL4*	This study	N/A
*UAS-Fur2-GFP#1*	This study	N/A
*UAS-Fur2-GFP#2*	This study	N/A
*UAS-Fur2-RFP*	This study	N/A
*UAS-fng-m1/2-GFP*	This study	N/A

Oligonucleotides		

See Table S2	This study	N/A

Software and algorithms		

ImageJ	NIH	RRID:SCR_003070
Photoshop	Adobe	RRID:SCR_014199
Imaris	Bitplan	RRID:SCR_007370
Metamorph	Molecular Devices	RRID:SCR_002368
Excel	Microsoft	RRID:SCR_016137
Prism	GraphPad	RRID:SCR_002798

## Highlights

- Fringe localizes at Golgi outposts in dendrites to promote branch retraction
- Furin 2 processes Fringe at the intraluminal domain at Golgi outposts
- Epidermal Delta induces non-canonical Notch signaling to suppress dendrite growth
- Fringe suppresses actin dynamics in dendrites and nociceptive response to heat
