# Supplementary material for: Fringe-positive Golgi outposts unite temporal Furin 2 convertase activity and spatial Delta signal to promote dendritic branch retraction
Source: Cell Rep. Author manuscript; Available in PMC 2024 Oct 9. (PMC11463699; doi:10.1016/j.celrep.2022.111372)
Supplement: 1 [file NIHMS1838957-supplement-1.pdf]

Figure S1

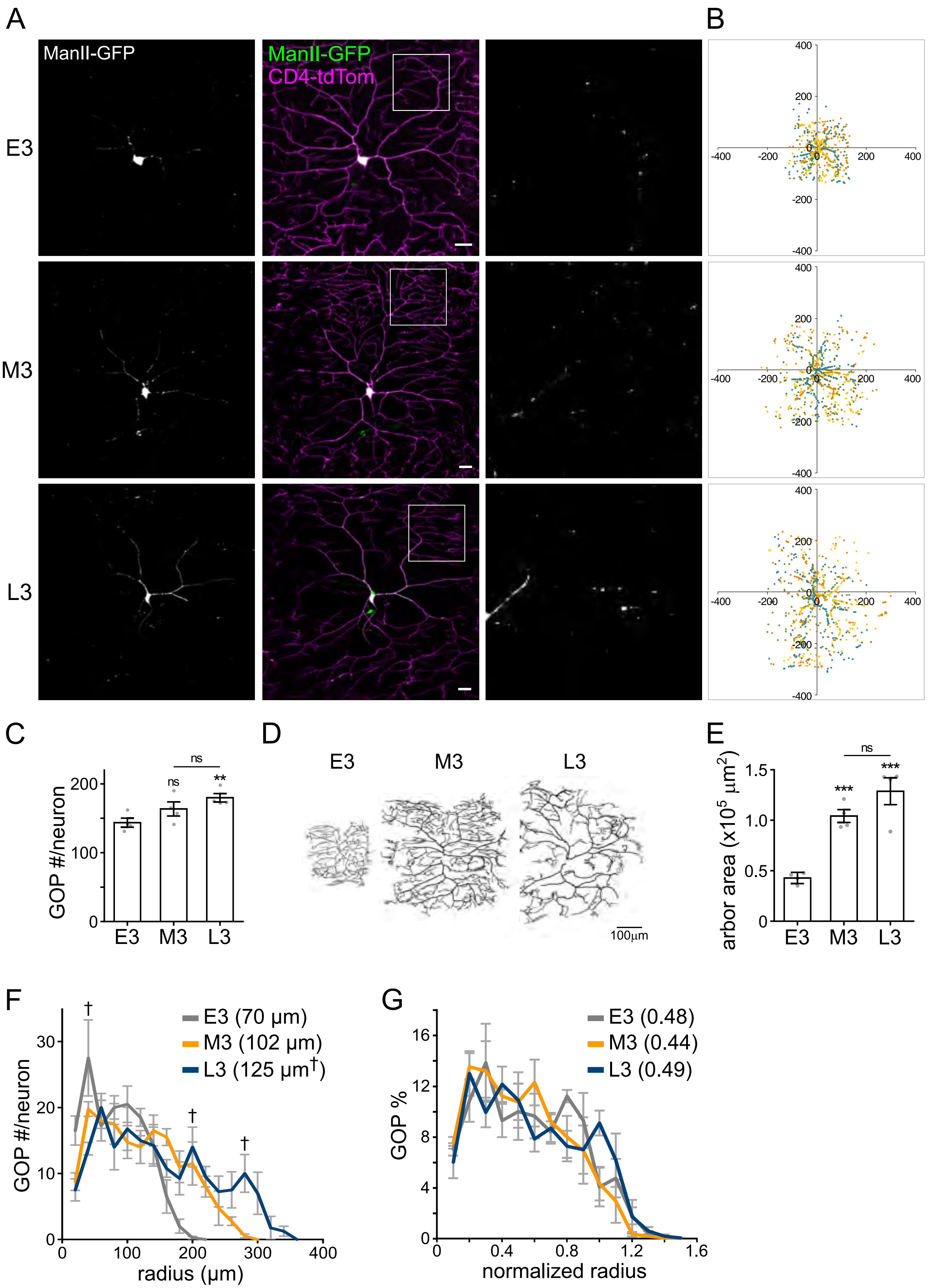

A
ManII-GFP
ManII-GFP
CD4-tdTom
E3
M3
L3
B
C
GOP #/neuron
ns
ns
**
E3 M3 L3
D
E3
M3
L3
100μm
E
arbor area (x10^5 μm^2)
ns
***
***
E3 M3 L3
F
E3 (70 μm)
M3 (102 μm)
L3 (125 μm†)
GOP #/neuron
radius (μm)
G
E3 (0.48)
M3 (0.44)
L3 (0.49)
GOP %
normalized radius

Figure S1. Constant GOP distribution in C4da dendrites at the E3, M3, and L3 stages.

(A) Distribution of ManII-GFP (white in the first and third columns, and green in the second column) driven by *ppk-GAL4* in C4da dendrites labeled by CD4-tdTom (magenta) at the E3, M3, and L3 stages. Boxed areas in the middle column are amplified in the right-most column for showing ManII-GFP puncta. (B) Scatter plots of the distributions of ManII-GFP puncta from C4da neurons (each coded with different colors) are superimposed in a 2D coordinate system with the somatic centroid as the origin, and the x- and y- axis (μm) representing anteroposterior and dorsoventral direction, respectively. (C) Numbers of ManII-GFP puncta (GOP) per neuron at the E3 (n=4), M3 (n=4), and L3 (n=4) stages. (D) Camera lucida of representative C4da dendritic arbors at the E3, M3, and L3 stages. (E) Averaged areas of dendritic fields at the E3 (n=4), M3 (n=4), and L3 (n=4) stages. Error bars represent S.E.M. Statistical significance was determined by Student's t test as ** for $p < 0.01$, *** for $p < 0.001$, and ns for not significant. (F, G) Concentric distribution curves for GOP at the E3 (gray, n=4), M3 (orange, n=4), and L3 (blue, n=4) stages show (F) mean numbers of GOP within 20 μm radial intervals, or (G) mean percentages of total puncta along the normalized radial axis from somatic centroid. Statistical significance was determined by two-way ANOVA with Bonferroni post-hoc test; † represent $p < 0.05$ in comparing E3 and L3. Averaged median radii of accumulative ManII-GFP distributions (F) or with normalization (G) are shown in parentheses for the E3, M3, and L3 stages. Statistical significance was determined by Student's t test with † represent $p < 0.05$ in comparing E3 and L3. Scale bars are 20 μm.

Figure S2

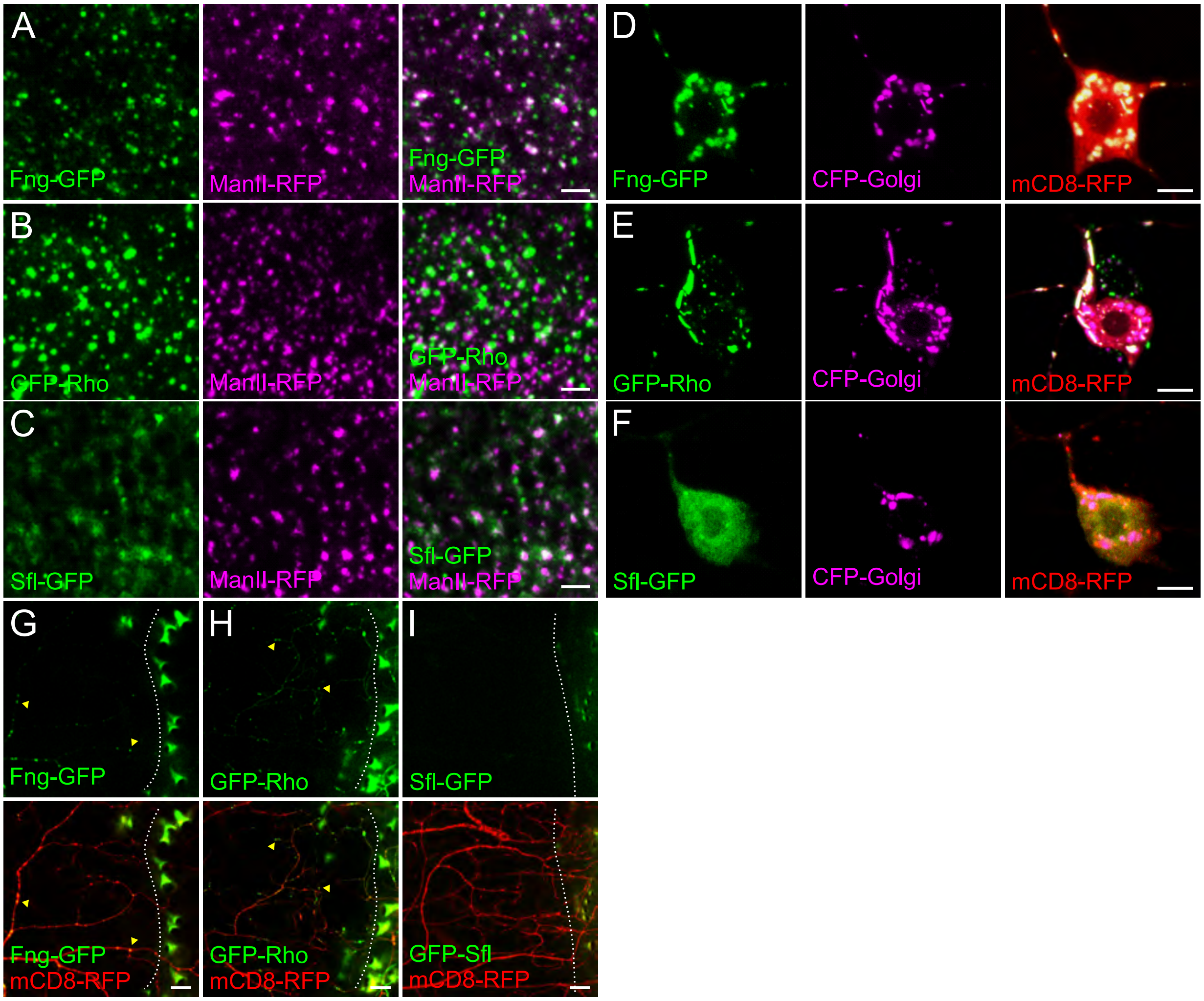

Figure S2. Differential localizations of Fng, Rho, and Sfl in Golgi fragments.

(A-C) Localization of (A) Fng-GFP, (B) GFP-Rho and (C) Sfl-GFP proteins (green) to ManII-RFP-positive Golgi fragments (magenta) expressed by *ms1096-GAL4* in wing disc cells. (D-F) Localization of (D) Fng-GFP, (E) GFP-Rho or (F) Sfl-GFP proteins (green) to CFP-Golgi fragments (magenta) expressed by *ppk-GAL4* in C4da neurons with mCD8-RFP-labeled dendrites. (G-I) Localization of (G) Fng-GFP, (H) GFP-Rho or (I) Sfl-GFP proteins (green) in C4da dendrites (red) expressed by *ppk-GAL4*. Fng-GFP or GFP-Rho puncta are indicated (arrow heads). Autofluorescence signals at the right (to white dashed lines) were from epidermal denticles. Scale bars are 5 μm in (A-F) and 20 μm in (G-I).

Figure S3

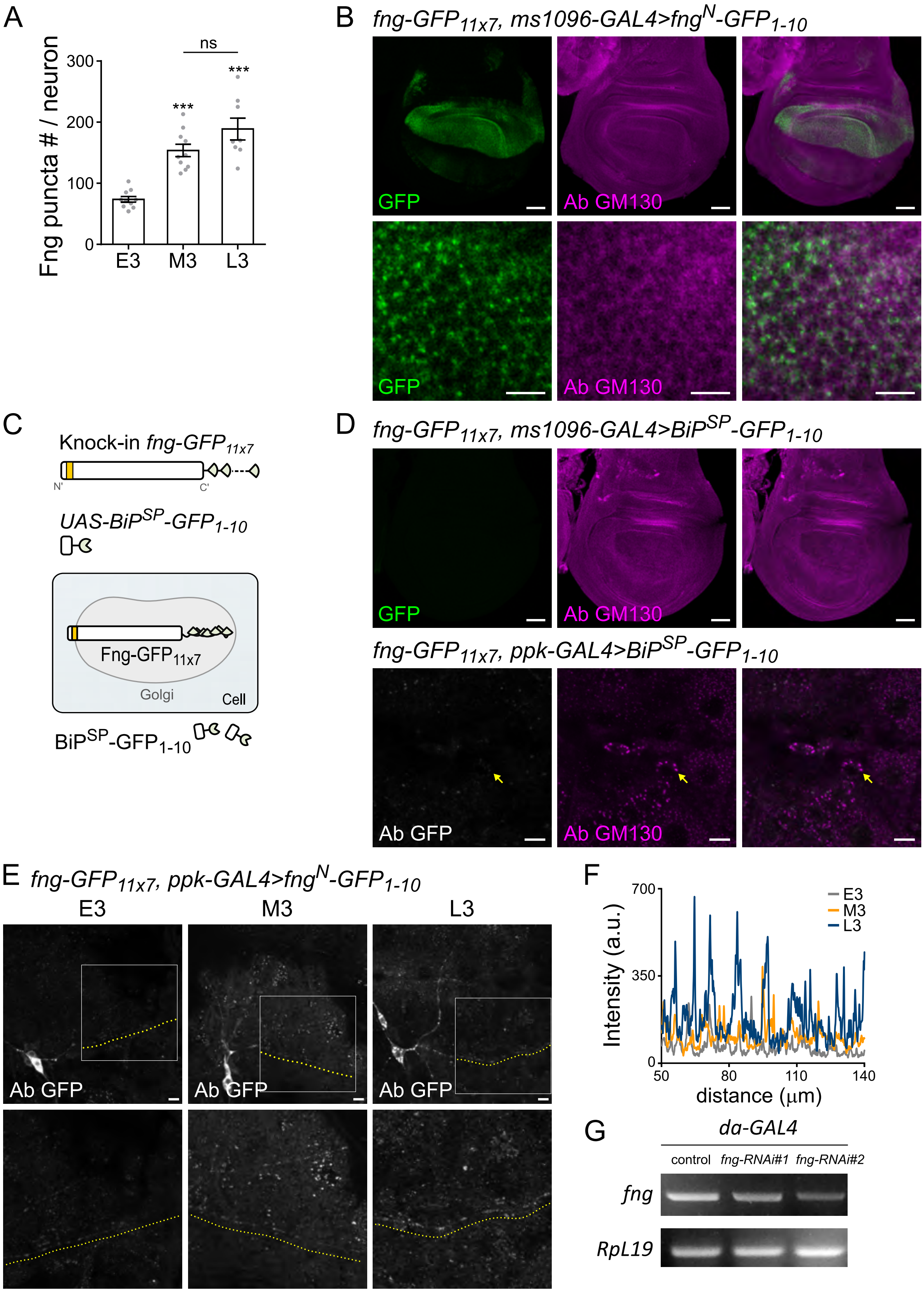
A
ns
300
200
100
0
***
***
Fng puncta # / neuron
E3
M3
L3
B
fng-GFP11x7, ms1096-GAL4>fngN-GFP1-10
GFP
Ab GM130
GFP
Ab GM130
C
Knock-in fng-GFP11x7
N'
C'
UAS-BiPSP-GFP1-10
Fng-GFP11x7
Golgi
Cell
BiPSP-GFP1-10
D
fng-GFP11x7, ms1096-GAL4>BiPSP-GFP1-10
GFP
Ab GM130
fng-GFP11x7, ppk-GAL4>BiPSP-GFP1-10
Ab GFP
Ab GM130
E
fng-GFP11x7, ppk-GAL4>fngN-GFP1-10
E3
M3
L3
Ab GFP
Ab GFP
Ab GFP
F
700
350
0
Intensity (a.u.)
E3
M3
L3
50
80
110
140
distance (μm)
G
da-GAL4
control
fng-RNAi#1
fng-RNAi#2
fng
RpL19

Figure S3. Increased Fng in the dendrites of C4da neurons at the M3 and L3 stages.

(A) Numbers of Fng-GFP puncta per neuron in E3 (n=10), M3 (n=10), and L3 (n=8) stages. Error bars represent S.E.M. Statistical significance was determined by Student's t test as *** for $p < 0.001$ and ns for not significant. (B) Schematics for knock-in *fng-GFP$_{11x7}$* and GAL4-driven *UAS-BiP^{SP}-GFP$_{1-10}$*. Fng-GFP$_{11x7}$ is tethered to Golgi with transmembrane domains (yellow), and the BiP^{SP}-GFP$_{1-10}$ is secreted extracellularly. No GFP is reconstituted from Fng-GFP$_{11x7}$ and BiP^{SP}-GFP$_{1-10}$ in the Golgi lumen. (C) With *ms1096-GAL4*-driving *UAS-fng^{N}-GFP$_{1-10}$* expression in the *fng-GFP$_{7x11}$* knock-in larvae, reconstitute GFP signals (green) from Fng-GFP$_{11x7/1-10}$ were detected in dorsal wing pouch (top row). Localization at the Golgi structures immuno-labeled by GM130 (magenta) was shown in magnified images (bottom row). (D) In *fng*-GFP$_{11x7}$ knock-in larvae, BiP^{SP}-GFP$_{1-10}$ was driven by *ms1096-GAL4* (top row) or *ppk-GAL4* (bottom row). No detectable reconstituted GFP signals were in wing disc cells, and no detectable reconstituted GFP signals immunostained by GFP antibody (Ab GFP) in C4da neurons. (E) GFP signals from reconstituted Fng-GFP$_{11x7/1-10}$ (white) were detected by GFP antibody immunostaining (Ab GFP) in C4da dendrites at the E3, M3 and L3 stages, while Fng^{N}GFP$_{1-10}$ was driven by *ppk-GAL4*. Dendritic regions beyond proximal 50 μm dendritic segments (white square) were enlarged (bottom row). (F) Intensities of GFP signals along the dendritic segments of 90 μm in length (above yellow dashed lines in (E)) were shown for the E3 (gray), M3 (orange) and L3 (blue) stages. Respective background signals were subtracted. (G) *UAS-fng-RNAi#1* and *UAS-fng-RNAi#2* were driven by *da-GAL4* ubiquitously in larvae, and the mRNA levels of *fng* and the *RpL19* control were detected by RT-PCR. Scale bars are 50 μm in (B, D top rows) and 10 μm in (B, D bottom rows, E).

Figure S4

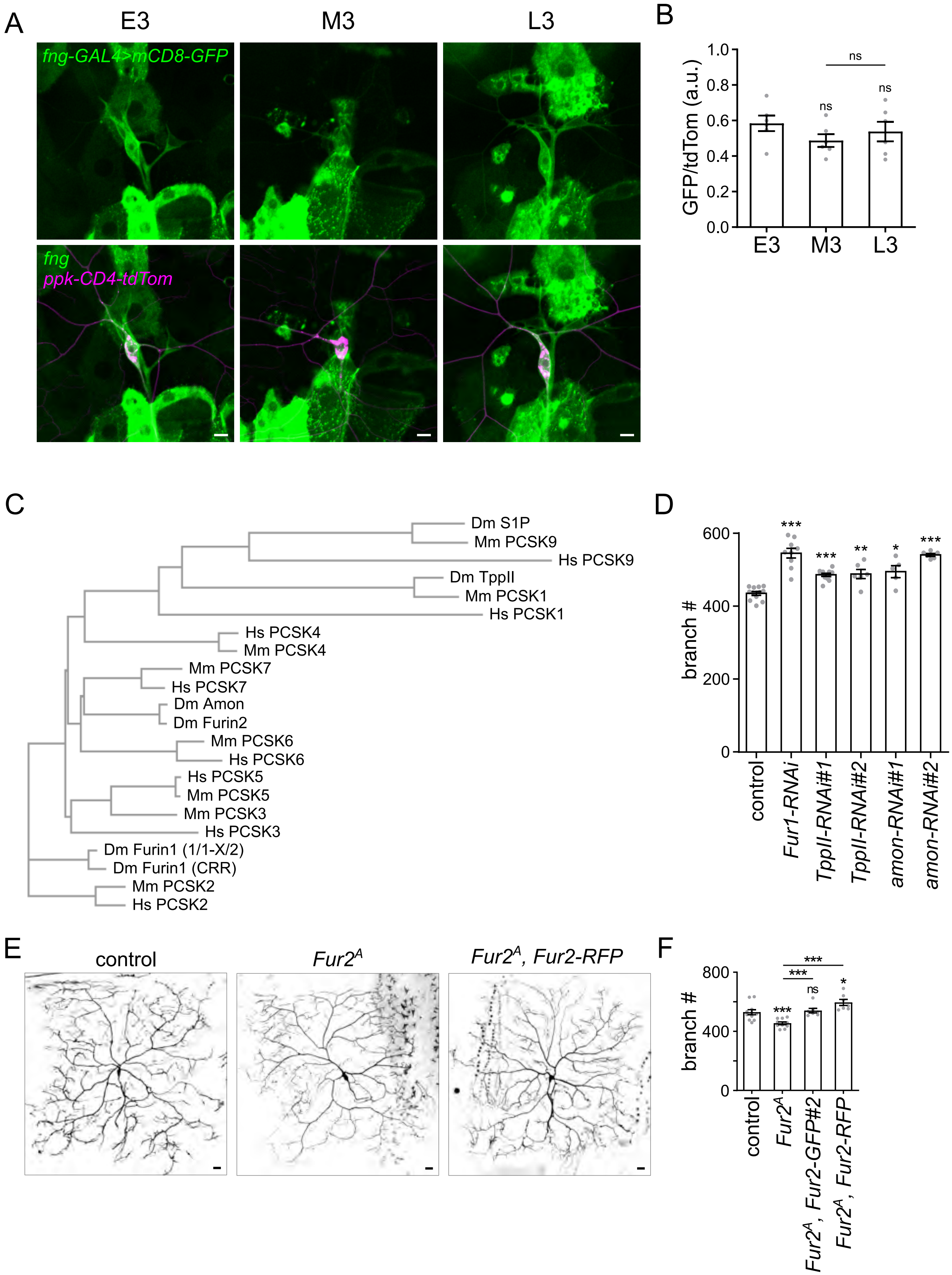

A
E3
M3
L3
fng-GAL4>mCD8-GFP
fng
ppk-CD4-tdTom
B
GFP/tdTom (a.u.)
1.0
0.8
0.6
0.4
0.2
0.0
ns
ns
ns
E3
M3
L3
C
Dm S1P
Mm PCSK9
Hs PCSK9
Dm TppII
Mm PCSK1
Hs PCSK1
Hs PCSK4
Mm PCSK4
Mm PCSK7
Hs PCSK7
Dm Amon
Dm Furin2
Mm PCSK6
Hs PCSK6
Hs PCSK5
Mm PCSK5
Mm PCSK3
Hs PCSK3
Dm Furin1 (1/1-X/2)
Dm Furin1 (CRR)
Mm PCSK2
Hs PCSK2
D
branch #
600
400
200
0
***
***
**
*
***
control
Fur1-RNAi
TppII-RNAi#1
TppII-RNAi#2
amon-RNAi#1
amon-RNAi#2
E
control
Fur2^A
Fur2^A, Fur2-RFP
F
branch #
800
400
0
***
***
ns
***
*
control
Fur2^A
Fur2^A, Fur2-GFP#2
Fur2^A, Fur2-RFP

Figure S4. Comparable *fng-GAL4* expression levels in C4da neurons at the E3, M3, and L3 stages, and effects of PCSK knockdowns on dendritic arborization of C4da neurons.

(A) Expression of *UAS-mCD8-GFP* driven by *fng-GAL4* (green) was detected in C4da neurons labeled by *ppk-CD4-tdTom* (magenta, bottom row) at the E3, M3, and L3 stages. (B) Bar graph showing mCD8-GFP intensities normalized against tdTom intensities within C4da soma at the E3 (n=6), M3 (n=6), and L3 (n=6) stages, shown as arbitrary units (a.u.). (C) Phylogenetic tree of the PCSK proteins in *Drosophila melanogaster* (Dm), *Mus musculus* (Mm) and *Homo sapiens* (Hs). (D) Total terminal branches quantification of C4da neurons subjected to *Fur1-RNAi* (n=9), *TppII-RNAi#1* (n=12), *TppII-RNAi#2* (n=6), *amon-RNAi#1* (n=5), or *amon-RNAi#2* (n=6) knockdown driven by *ppk-GAL4*, with *ppk-GAL4* (from 3M) as a control. (E) C4da MARCM clones of control and $Fur2^{A}$, and $Fur2^{A}$ with *Fur2-GFP#2* or *Fur2-RFP*. (F) Quantification of total terminal branches for C4da MARCM clones of control (n=11), $Fur2^{A}$ (n=10), and $Fur2^{A}$ with *Fur2-GFP#2* (n=7) or *Fur2-RFP* (n=7). Error bars represent S.E.M. Statistical significance was determined by Student's t test and shown as *$p < 0.05$, **$p < 0.01$, *** $p < 0.001$, and ns for not significant. Scale bar are 20 μm.

Figure S5

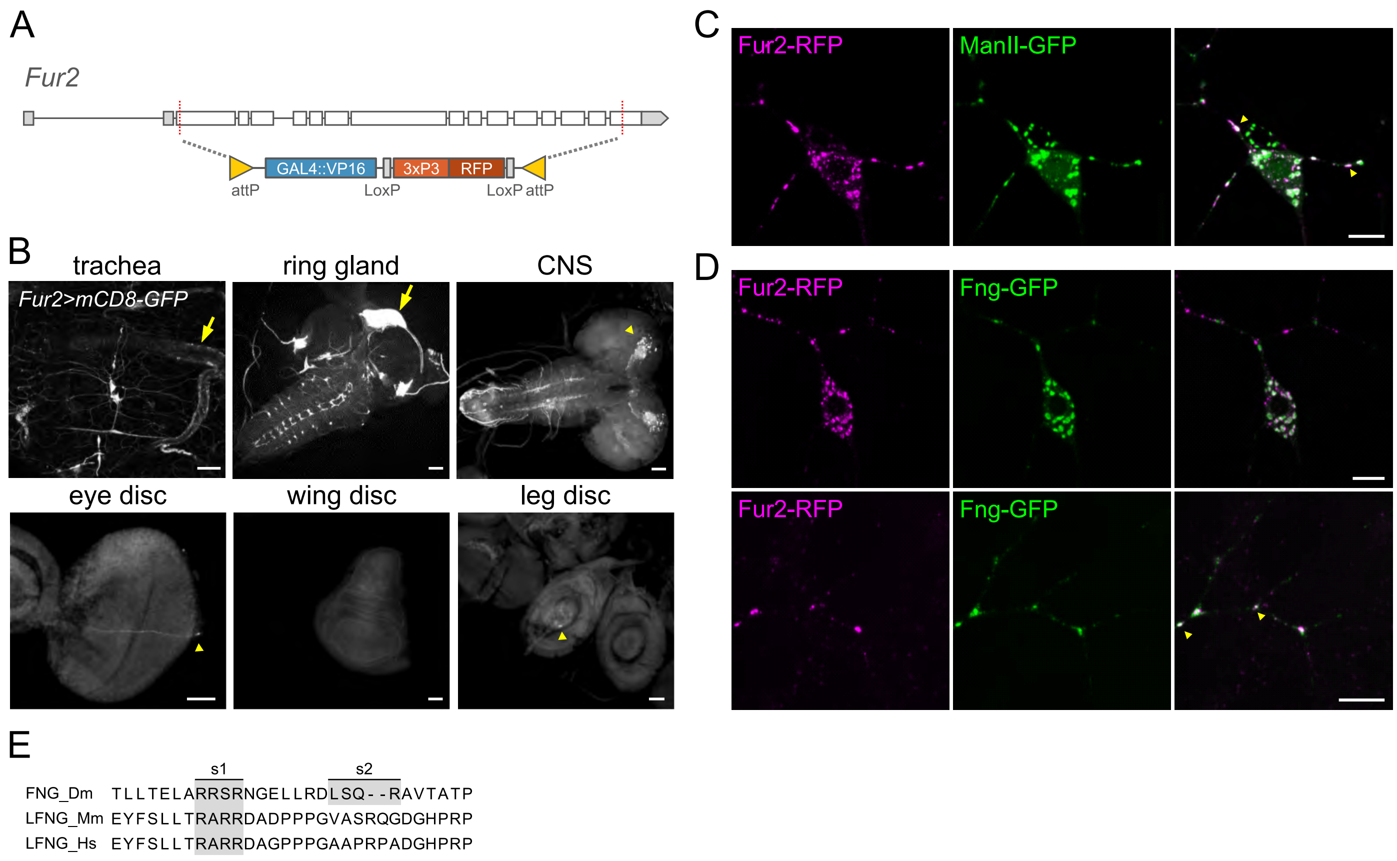

Figure S5. Fur2 expression and localization.

(A) Schematic illustration of the genomic structure of *Fur2* and the generation of *Fur2-GAL4* knock-in. The *Fur2* coding region is replaced with *GAL4::VP16* and *3xP3-RFP* marker, which was removed by LoxP recombination. *Fur2-GAL4* homozygotes are embryonically lethal. (B) mCD8-GFP driven by *Fur2-GAL4* was observed in the trachea (arrow), ring gland (arrow), and neurons in the brain, ventral nerve cord, and imaginal discs (arrowheads) at larval stages. (C) Fur2-RFP (magenta) and ManII-GFP (green) co-expressed by *ppk-GAL4* co-localize in the soma and dendrites (arrowheads). (D) Fur2-RFP (magenta) and Fng-GFP (green) co-expressed by *ppk-GAL4* display co-localization of signals in the soma and dendrites (arrowheads). (E) Conserved s1 proteolytic site (shaded) for PCSK proteins in fly Fng and mammalian Lunatic fringe (LFNG) and s2 site in fly Fng. Scale bars are 50 µm in (B) and 10 µm in (C, D).

Figure S6

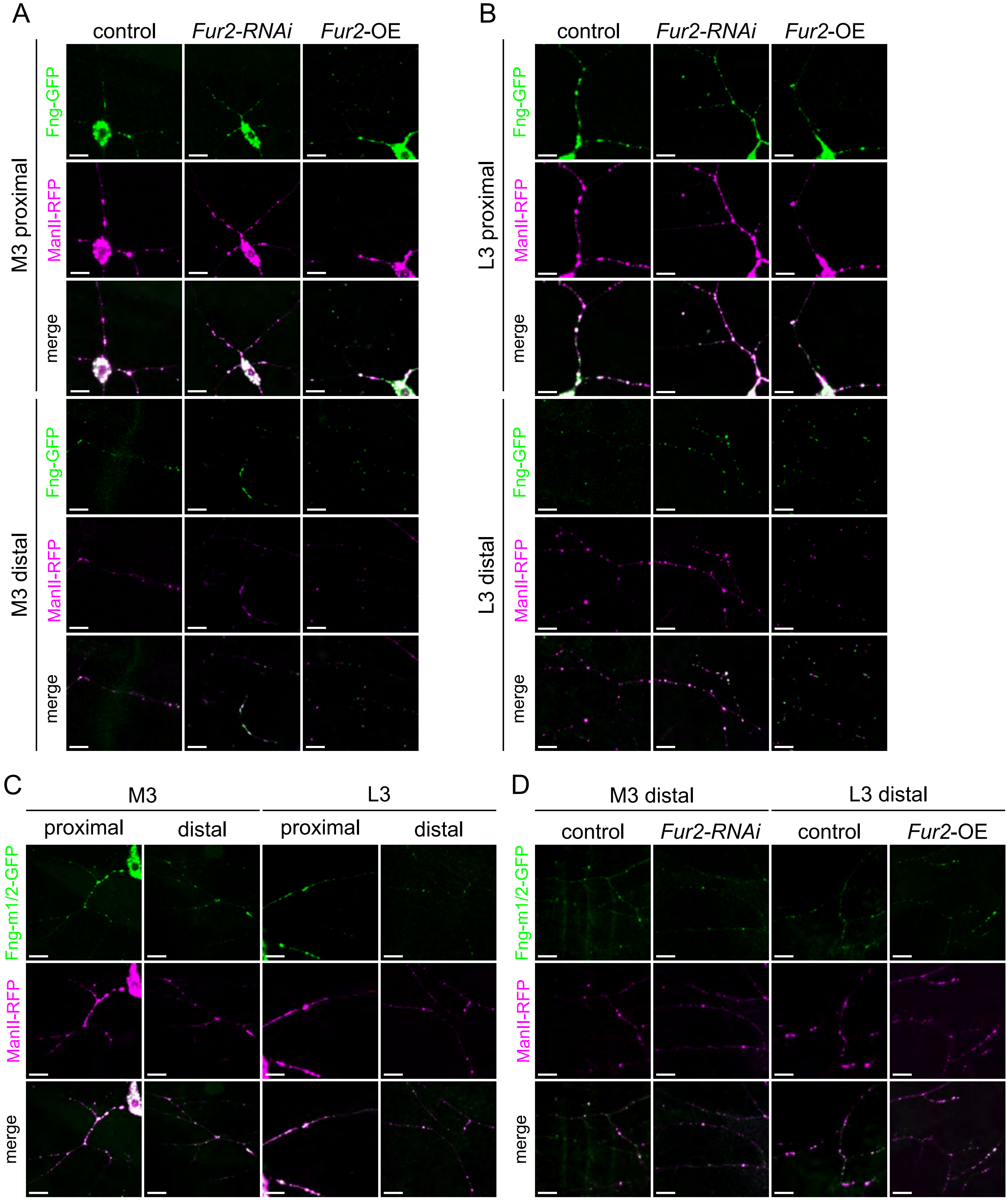

A
control
Fur2-RNAi
Fur2-OE
M3 proximal
M3 distal
Fng-GFP
ManII-RFP
merge
B
L3 proximal
L3 distal
C
M3
L3
proximal
distal
Fng-m1/2-GFP
D
M3 distal
L3 distal

Figure S6. Separated and merged fluorescent emissions of Fng or Fng-m1/2 and GOP reporters.

(A-B) Fng-GFP (green) and GOP marker ManII-RFP (magenta), showing in proximal or distal dendrites, were co-overexpressed by *ppk-GAL4* in control (also co-expressing *lacZ*), *Fur2-RNAi* knockdown, or *Fur2* overexpression (*Fur2*-OE) C4da neurons at the (A) M3 and (B) L3 stages (merged images also show in Figures 4F and 4H). (C-D) Fng-m1/2-GFP (green) and ManII-RFP (magenta), showing in proximal or distal dendrites, were co-overexpressed by *ppk-GAL4* in C4da neurons (C) at the M3 and L3 stages, or in (D) control (also co-expressing *lacZ*), M3 *Fur2-RNAi* knockdown, and L3 *Fur2* overexpression (*Fur2*-OE) C4da neurons (merged images also show in Figures 5C and 5E). Scale bars are 10 μm.

Figure S7

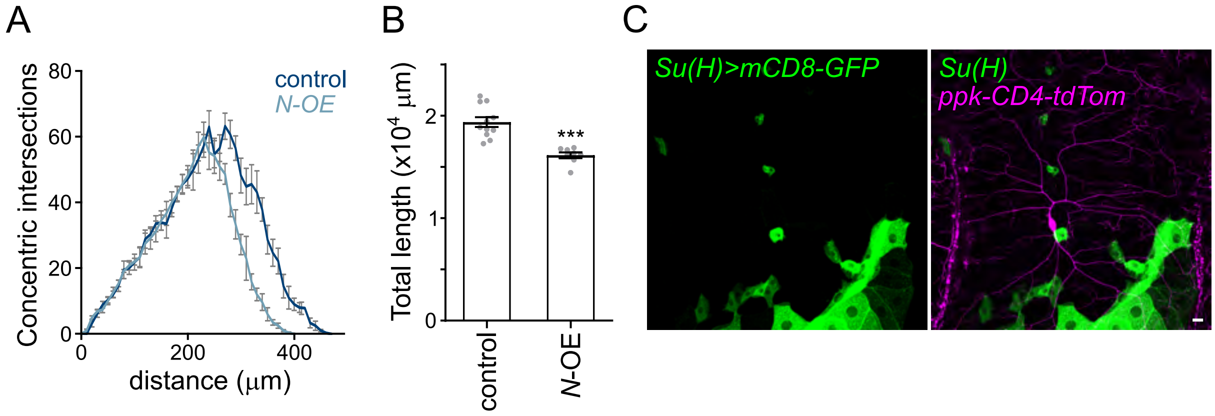

Figure S7. Suppression of dendritic arborization upon *N* overexpression.

(A) Sholl analysis and (B) quantification of total dendritic length for the C4da dendrites in *ppk-GAL4* control (n=11) and *N* overexpression (*N*-OE) (n=8) neurons. (C) Representative image of mCD8-GFP expression (green) driven by *Su(H)-GAL4,* which was not detected in C4da neurons labeled by CD4-tdTom (magenta). Error bars represent S.E.M., and comparisons between data groups were processed by Student's t test with significance shown as ***p < 0.001. Scale bar is 20 μm.

Table S2. Primers used in this study. Related to Figures 2, 4, 5, S3, S4, S5.

	primer	Restriction enzyme site
fng^{N} (f)	GAATTCATG ATGAGCCTGACTGTGCT	EcoRI
fng^{N} (re)	CTCGAGGCTGTGCGGCACTTGGATG	XhoI
GFP$_{1\text{-}10}$ (f)	CTCGAGATGTCCAAAGGAGAAGAACTGTTT	XhoI
GFP$_{1\text{-}10}$ (re)	TCTAGATTTTTCATTTGGATCTTTGCTCA	XbaI
BiP^{SP} (f)	CGTCAGAATTGATCTAGAATTCATGAAGTTATGC ATATTACTGGCCGTCGTGGCCTTTGTTGGCC-3’	EcoRI
BiP^{SP} (f-2)	GGCCTTTGTTGGCCTCTCGCTCGGGCTCGAGGT G-3’	XhoI
BiP^{SP} (re)	CAAAGATCCTCTAGAGGTACCTCAAATGCATTCG CCGGGATTCTGGG	XbaI
fng RT-PCR (f)	CCAAAGCAATCCTGGTCATT	
fng RT-PCR (re)	GGCAGTGCAGGGAGTAGAAG	
Fur2 (f)	ATGTCAAATACGACGAGGTCCAGC	
Fur2 (re)	GCTACGGATGTTTGTACGGC	
fng point mutation		
fng-m1 (f)	GCTAACAGAATTGGCCGCAAGGAGCGCGAATGG GGAACTG	
fng-m1 (re)	CAGTTCCCCATTCGCGCTCCTTGCGGCCAATTC TGTTAGC	
fng-m2 (f)	CTGCTCCGCGATGCGTCCCAAGCAGCGGTGAC GG	
fng-m2 (re)	CCGTCACCGCTGCTTGGGACGCATCGCGGAGC AG	
